# Comparative Transcriptomic Analysis of Rhinovirus and Influenza Virus Infection

**DOI:** 10.3389/fmicb.2020.01580

**Published:** 2020-07-21

**Authors:** Thrimendra Kaushika Dissanayake, Sascha Schäuble, Mohammad Hassan Mirhakkak, Wai-Lan Wu, Anthony Chin-Ki Ng, Cyril C. Y. Yip, Albert García López, Thomas Wolf, Man-Lung Yeung, Kwok-Hung Chan, Kwok-Yung Yuen, Gianni Panagiotou, Kelvin Kai-Wang To

**Affiliations:** ^1^Department of Microbiology, Li Ka Shing Faculty of Medicine, The University of Hong Kong, Hong Kong, China; ^2^Systems Biology and Bioinformatics Unit, Leibniz Institute for Natural Product Research and Infection Biology – Hans Knöll Institute, Jena, Germany; ^3^State Key Laboratory for Emerging Infectious Diseases, Li Ka Shing Faculty of Medicine, The University of Hong Kong, Hong Kong, China; ^4^Department of Clinical Microbiology and Infection Control, The University of Hong Kong, Hong Kong, China; ^5^Carol Yu Centre for Infection, The University of Hong Kong, Hong Kong, China; ^6^Systems Biology and Bioinformatics Group, School of Biological Sciences, Faculty of Sciences, The University of Hong Kong, Hong Kong, China

**Keywords:** influenza, rhinovirus, transcriptomics analysis, ICAM5, interferons, cytokines

## Abstract

Rhinovirus (RV) and influenza virus are the most frequently detected respiratory viruses among adult patients with community acquired pneumonia. Previous clinical studies have identified major differences in the clinical presentations and inflammatory or immune response during these infections. A systematic transcriptomic analysis directly comparing influenza and RV is lacking. Here, we sought to compare the transcriptomic response to these viral infections. Human airway epithelial Calu-3 cells were infected with contemporary clinical isolates of RV, influenza A virus (IAV), or influenza B virus (IBV). Host gene expression was determined using RNA-seq. Differentially expressed genes (DEGs) with respect to mock-infected cells were identified using the overlapping gene-set of four different statistical models. Transcriptomic analysis showed that RV-infected cells have a more blunted host response with fewer DEGs than IAV or IBV-infected cells. IFNL1 and CXCL10 were among the most upregulated DEGs during RV, IAV, and IBV infection. Other DEGs that were highly expressed for all 3 viruses were mainly genes related to type I or type III interferons (RSAD2, IDO1) and chemokines (CXCL11). Notably, ICAM5, a known receptor for enterovirus D68, was highly expressed during RV infection only. Gene Set Enrichment Analysis (GSEA) confirmed that pathways associated with interferon response, innate immunity, or regulation of inflammatory response, were most perturbed for all three viruses. Network analysis showed that steroid-related pathways were enriched. Taken together, our data using contemporary virus strains suggests that genes related to interferon and chemokine predominated the host response associated with RV, IAV, and IBV infection. Several highly expressed genes, especially ICAM5 which is preferentially-induced during RV infection, deserve further investigation.

## Introduction

Influenza virus is well-known to cause severe respiratory tract infection. The number of deaths associated with seasonal influenza virus infection has been estimated to be between 290,000 and 650,000 globally (Iuliano et al., 2018). Influenza pandemics and seasonal epidemics are associated with sudden surge in hospitalizations and deaths. Unlike influenza virus, rhinovirus (RV) has been relatively neglected because of the deep-rooted association with common cold. However, recent studies have shown that RV is a frequent cause of severe respiratory illnesses and is associated with a higher case-fatality rate than influenza virus infection (Jain et al., 2015; Hung et al., 2017; Ieven et al., 2018; Prill et al., 2018). RV is also the most commonly detected respiratory viruses among patients with lower respiratory tract infection. Outbreaks of severe RV infection have been reported (Marcone et al., 2017).

Clinically, both influenza virus and RV cause respiratory tract infection, and can be complicated by extrapulmonary disease (To et al., 2016a, 2019). However, there are important clinical differences between RV and influenza virus infection. We have previously found that hospitalized patients with RV infection are more likely to present with exacerbation of chronic lung diseases or complicated with extrapulmonary manifestations (To et al., 2018, 2019).

Host response to infection provides tremendous insights into the pathogenesis of an infection. Our previous study showed that a persistently dysregulated cytokine and chemokine response was associated with severe influenza A(H1N1)pdm09 infection (To et al., 2010). Our study in adult hospitalized patients showed that influenza virus was associated with a much more robust cytokine and chemokine response, especially CXCL10. In contrast, RV was associated with an exaggerated T_*H*_2 response, characterized by an elevated eosinophil count and IL-5 (To et al., 2018). The avian influenza virus A(H7N9), which is associated with a much higher case-fatality rate than seasonal influenza virus, can directly infect human mononuclear cells and induce much more heightened cytokine response than seasonal influenza virus (Lee et al., 2017). Host gene expression has been utilized to assess host response. Peripheral blood gene expression studies have shown that influenza and RV patients exhibit different gene expression profile (Zaas et al., 2009; Zhai et al., 2015).

Since the primary site of damage occurs in the lung, the local host response also plays an important role in respiratory virus infection. Host gene response after RV infection has been previously compared to that of influenza virus infection in a human bronchial epithelial cell line BEAS-2B cells using microarray analysis (Kim et al., 2015). However, BEAS-2B has high basal production of interferon-stimulated genes which may affect the response of other host genes (Seng et al., 2014; Hillyer et al., 2018). In this study, we compared the host response between influenza virus and RV in a well-characterized lower airway cell line, Calu-3, using RNA-seq. Calu-3 cell line was chosen because it supports the growth of both influenza and RV (Rajan et al., 2013, 2014; To et al., 2016b). Furthermore, Calu-3 cell line has been used extensively for transcriptomic experiments previously because of high reproducibility (Aevermann et al., 2014; Menachery et al., 2014).

## Materials and Methods

### Viruses

The viruses used in this study were isolated from patients in Hong Kong. Influenza A(H1N1) virus A/HK/415742/2009 and influenza B virus (IBV) B/HK/411989/2011 were used in our previous studies (Zheng et al., 2010; To et al., 2016a). RV 451892/2011 was isolated from a patient with pneumonia, and belongs to RV species A type 1A. Influenza A virus (IAV) and IBV were propagated in Madin Darby canine kidney (MDCK) cells at 37°C, while RV was propagated in RD cells at 33°C. Viruses were concentrated by ultracentrifugation and then resuspended in 1 ml of minimum essential medium (MEM) and Dulbecco’s Modified Eagle Medium (DMEM) for influenza virus and RV, respectively. Plaque number was determined on MDCK cells for IAV and IBV, and on H1HeLa cells (ATCC CRL-1958) for RV.

### Immunofluorescence Assay for Viral Protein Expression

Immunofluorescence assay for viral protein expression was performed as described previously with modifications (To et al., 2016). Briefly, IAV, IBV or RV was added to Calu-3 cells at 1 multiplicity of infection (MOI) and incubated at 37°C for 1 h. At 1 h post infection, cells were washed and minimum essential free medium was added. At 24 h post-infection, the seeded cells were fixed in chilled acetone at −20°C for 10 min and stained with fluorescein-tagged murine monoclonal antibodies against IAV, IBV (IAV and IBV DFA Reagent, D3^®^ Ultra 8TM DFA Respiratory Virus Screening and Identification Kit, Diagnostic Hybrids, Inc., Quidel, United States) or pan-picornavirus proteins (LIGHT DIAGNOSTICS^TM^ Pan-Enterovirus Reagent, Chemicon International, Temecula, CA, United States) at 37°C for 30 min and examined under fluorescence microscope.

### Virus Replication in Calu-3 Cells

Virus infection in Calu-3 cells (ATCC^®^ HTB-55^TM^, passage number: 7) was performed as we described previously with modification (To et al., 2009). Calu-3 was infected with IAV, IBV, and RV at 1 MOI in DMEM-F12 medium. For the determination of MOI, the virus quantification was performed using plaque assay on MDCK cells for IAV and IBV, and H1Hela for RV. Culture supernatant was collected at pre-determined time points and real-time reverse transcription quantitative polymerase chain reaction (RT-qPCR) for each virus was performed. RNA extraction and RT-qPCR were performed as we described previously with modifications (Zhao et al., 2018; Chan et al., 2019). Briefly, viral RNA was extracted using QIAamp^®^ Viral RNA Mini Kit (Qiagen, Hilden, Germany). One step RT-qPCR was performed using AgPath-ID^TM^ One-Step RT-PCR kit (Applied Biosystems, California, United States). The primers and probes for the detection of IAV, IBV, and RV were described previously with modifications (Supplementary Table S1; To et al., 2017, 2018). Real-time RT-qPCR was performed using LightCycler^®^ LC96 instrument (Roche, Mannheim, Germany). Duplicates were performed for each virus for each time point in two independent experiments. Statistical significance was calculated with two-way ANOVA.

### Real-Time RT-PCR for Cytokines, Chemokines, and ICAM5

Total RNA was extracted from infected cells using Qiagen RNeasy Mini Kit (Qiagen, Hilden, Germany). Real time reverse transcription polymerase chain reaction (RT-PCR) was performed as described previously with modifications (Wei et al., 2016). Briefly, RNA was reverse-transcribed to cDNA using PrimeScript^TM^ RT reagent kit (Takara Bio Inc., Shiga, Japan). Real time PCR assays for TNF-α, IL6, CXCL10, IFNβ, and ICAM5 were performed in LightCycler 96 system (Roche Applied Sciences, Indianapolis, United States) using primers and probes, and cycling condition as in Supplementary Table S2. The expression of house-keeping gene GAPDH was quantified in parallel for RNA normalization. The relative expression of the target genes was calculated by the ΔΔ Ct method. Statistical analysis was performed using PRISM^®^ 6.0 for Windows. Duplicates were performed for each virus at each time point in two independent experiments for measuring cytokine, chemokine and ICAM5 expression. Statistical significance was calculated with two-way ANOVA. One-way ANOVA with Holm-Sidak’s multiple comparisons was performed when comparing the fold change for ICAM5 expression in cells infected by different viruses or mock-infected cells test (^∗∗^*P* < 0.001, ^∗∗∗^*P* < 0.0001).

### Enzyme-Linked Immunosorbent Assay (ELISA) for Cytokines and Chemokines

Cell supernatant was collected from Calu-3 cell infected with IAV, IBV, and RV at 0, 6, 12, and 24 hpi in triplicates from 1 independent experiment. ELISA was done using Human DuoSet ELISA kits for IFN-λ1/λ2/λ3 (Catalog number DY7246, DY1587, DY5259) and CXCL10 (Catalog number DY266) (R&D Systems). Error bars represent SEM. Statistical significance was calculated with two-way ANOVA. Optical density for each well was measured at 450 nM (corrected for 570 nM during analysis) using Beckman Coulter DTX880 Multimode Detector.

### RNA-Seq Library Preparation, Sequencing, and Analysis

Total RNA was extracted from two replicates for mock infection (control), RV, IAV, and IBV for time points 0, 6, 12, and 24 h post infection, respectively, using RNeasy (Qiagen Hilden, Germany). RNA quantity and quality were assessed using NanoDrop Spectrophotometer and Bioanalyzer. Library preparation and Illumina sequencing (paired-end sequencing of 101 bp) were performed at University of Hong Kong, Centre for Genomic Sciences (HKU, CGS). cDNA libraries were prepared by KAPA Stranded mRNA-Seq Kit. One microgram of total RNA was used as starting material. Manufacturer’s protocol was followed. In brief, poly-A containing mRNA was collected by using poly-T oligo-attached magnetic beads. The purified mRNA was broken down into short fragments and was applied as template to synthesize the first-strand cDNA by using random hexamer-primer and reverse transcriptase. In the second strand cDNA synthesis, the mRNA template was removed and a replacement strand was generated to form the blunt-end double-stranded (ds) cDNA. The ds cDNA underwent 3′ adenylation and indexed adaptor ligation. The adaptor-ligated libraries were enriched by 10 cycles of polymerase chain reaction (PCR). The libraries were denatured and diluted to optimal concentration and applied in the cluster generation steps. HiSeq PE Cluster Kit v4 with cbot was used for cluster generation on the flow cell. Illumina HiSeq SBS Kit v4 was used for paired-end 101 bp sequencing. Whole dataset has been deposited in the NCBI Sequence Read Archive with accession number (PRJNA609228).

### Bioinformatics Analysis

RNA-seq raw data were processed following the GEO2RNA-Seq pipeline (Seelbinder et al., 2019) a RNA-Seq pre-processing workflow and package for analyzing read files, trimming of raw reads, mapping on reference genomes, counting reads per gene and finding significant differentially expressed genes (DEGs). Quality of raw read data was checked using FastQC version 0.11.5. Reads were quality- and adapter-trimmed using Trimmomatic version 0.36. Reads were rRNA-filtered using SortMeRNA version 2.1 with a single rRNA database concatenated from all rRNA databases shipped with SortMeRNA. Reads were mapped against the human reference genome and corresponding annotation GRCh38 89 (2017-05-07, obtained from Ensembl) using TopHat2 version 2.1.0.

Reads per feature (gene) were counted using Rsubread’s featureCounts version 1.20.6. The output off all pre-processing steps was reviewed using MultiQC version 1.1 (Supplementary Data S1). Additionally, human genome coverage and exome coverage per sample was calculated using R version 3.2.0 (Supplementary Data S2). Hierarchical clustering of MRN-normalized read counts using the unweighted pair group method with arithmetic mean (UPGM) metric was calculated with the “hclust” function from the R base package “stats” version 3.2.0. Principal component analysis (PCA) of MRN-normalized read counts was done with the “prcomp” function from the R base package “stats” version 3.2.0. DEGs were determined using four different tools, including DESeq (Anders and Huber, 2010), DESeq2 (Love et al., 2014), Limma (Ritchie et al., 2015), and EdgeR (Robinson et al., 2010). A gene was considered to be differentially expressed if the expression change was reported significantly different by all tools with a *p* ≤ 0.01. Using the consensus identification of DEGs by the aforementioned four tools assures controlling the false positive rate and increases the specificity at the expense of reduced sensitivity. DEGs were summarized together with log2 of fold changes based on MRN, TPM or RPKM normalization (Supplementary Data S3). MRN normalization was used for further analysis throughout the manuscript, since MRN was reported to be superior over other normalization schemes (Maza et al., 2013). Gene Set Enrichment Analysis (GSEA) (Subramanian et al., 2005) was performed by using g:Profiler (Raudvere et al., 2019). Enrichment score was calculated as the –log10 (*P*-value) as described previously (Morrison et al., 2014). Hierarchical clustering was performed for DEGs with an absolute log2 fold change greater than two using Ward’s method as implemented by the R package “pheatmap,” version 1.0.12.

## Results

### Virus Infection in Calu-3 Cells

**FIGURE 1:**
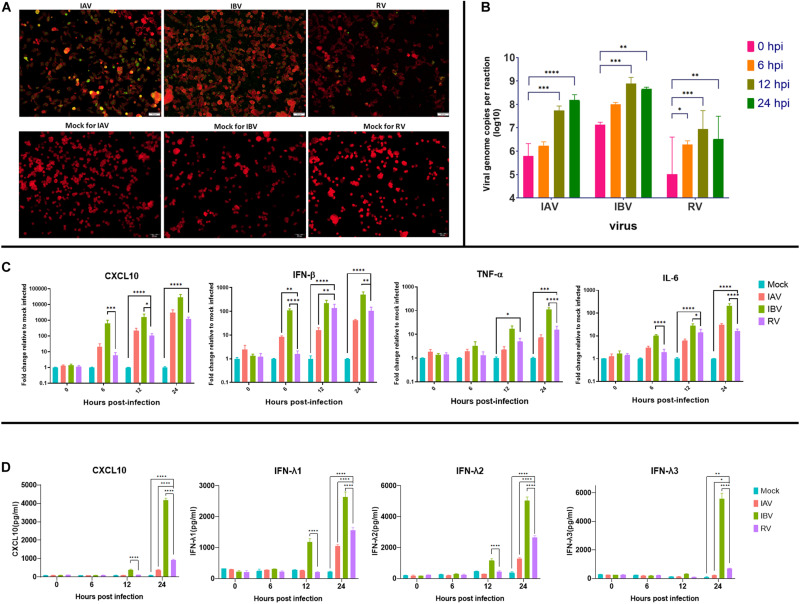
Infection of influenza A virus (IAV), influenza B virus (IBV) or rhinovirus (RV) in Calu-3 cells. **(A)** Calu-3 cells were infected with IAV, IBV, or RV at 1 MOI. IAV, IBV, and RV antigen expression was determined at 24 h post infection. Antigen expression was determined using fluorescein-tagged murine monoclonal antibodies against IAV, IBV, or RV. Mock-infected cells stained with respective monoclonal antibodies against IAV, IBV, or RV are shown in the bottom row. White scale bar = 50 μm. **(B)** Multicycle growth assay. Calu-3 cells were infected with IAV, IBV, or RV at 1 MOI. Viral load was determined using real-time RT-qPCR. **(C)** Cytokine and chemokine expression of Calu-3 cells infected with IAV, IBV, or RV at 1 MOI. Cytokine expression was determined using real time RT-PCR. GAPDH was used for normalization of gene expression. **(D)** Cytokine and chemokine protein expression of Calu-3 cells infected with IAV, IBV, or RV at 1 MOI. Protein expression was determined using ELISA. Bars **(B,C)** represent means (error bars show standard error of mean) of duplicates in two independent experiments. Bars **(D)** represent means (error bars show standard error of mean) of triplicates in one independent experiment. Statistical significance (for **B–D**) was calculated with two-way ANOVA. (^∗^*P*< 0.05, ^∗∗^*P* < 0.01, ^∗∗∗^*P* < 0.001, ^****^P < 0.0001). hpi, hours post infection; MOI, multiplicity of infection.

To confirm whether Calu-3 cells are susceptible to IAV, IBV, and RV infection, antigen expression and viral replication were determined. Immunofluorescence assay showed that IAV, IBV and RV could infect Calu-3 cells at 1 MOI (Figure 1A). Viral load assay showed that all three viruses could replicate in Calu-3 cells (Figure 1B). Next, cytokine and chemokine expression were measured to determine the host response of Calu-3 cells after IAV, IBV or RV infection. TNF-α, IL-6, CXCL10, and IFN-β were induced after infection with all three viruses (Figure 1C). ELISA for the IFN-λ1/λ2/λ3, and CXCL10 showed detection of proteins at 12 hpi for IBV and 24 hpi for IAV and RV (Figure 1D). Results shown in Figures 1C,D were consistent with RNA-seq data.

### Longitudinal RNA-Seq During Infection

RNA-seq was performed to determine the gene expression profile during IAV, IBV, and RV infection. Using software from Illumina (bcl2fastq), sequencing reads were assigned into individual samples with each sample having an average throughput of 6.6 Gb (Supplementary Table S3) and a total throughput of 210 Gb. In terms of sequence quality, an average of 93% of the bases achieved a quality score of Q30 where Q30 denotes the accuracy of a base call to be 99.9%. MultiQC and mapping statistics table showed very good assignment rates of over 85% reads assigned to the human reference genome for all samples (Supplementary Data S1, S2).

**FIGURE 2:**
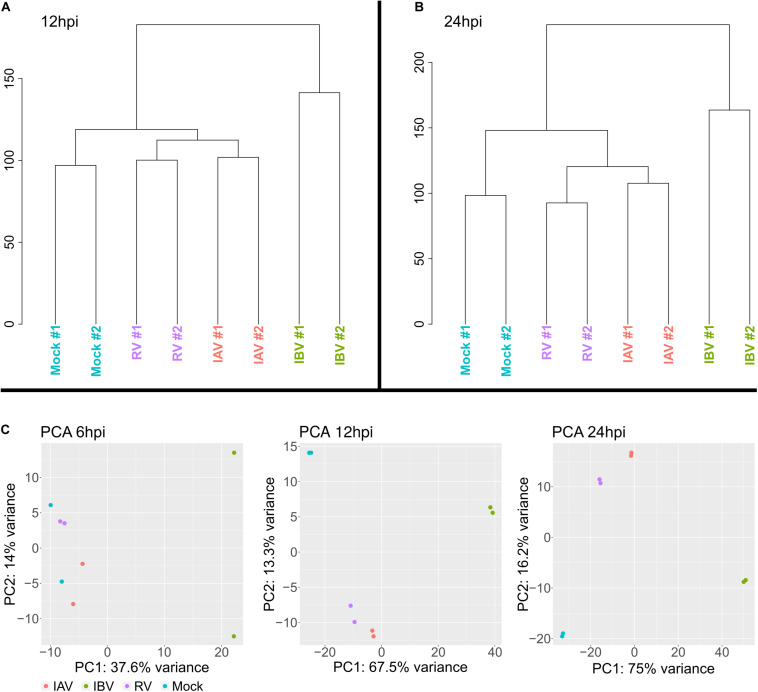
Hierarchical clustering of gene expression for **(A)** 12 hpi time point and **(B)** 24 hpi time point showing a distinct gene expression for IAV, IBV, and RV. **(C)** Principal component analysis for 6, 12, and 24 hpi.

A first global overview by hierarchical clustering of read counts showed a clear separation of the IAV, IBV, RV from- mock infection samples at 12 and 24 h post infection (hpi) (Figures 2A,B). While IBV showed already clear separation at 6 hpi, there was no clear separation between IAV, RV and mock-infected cells at 6 hpi, however (Figure 2C). Expression was notable for several mitochondrial genes and influenced explained variance in the PCAs (Supplementary Figure S1). In addition, several of these most influencing genes, e.g., *IFIT2*, are known to possess immune system functionality.

#### Differentially Expressed Genes (DEGs) Analysis

In order to elucidate the difference between RV and influenza virus infection, we compared the DEGs after RV, IAV, and IBV infection against mock and found a high agreement across all four tools used for DEG identification (Supplementary Figure S2). Of note, using the intersect of four different tools for identification of DEGs ensured high specificity, and thus reliability, at a tolerable sensitivity drop (Supplementary Figure S2). We observed a gradual increase in the number of significantly upregulated and downregulated DEGs for all three viral infections from 6 to 24 hpi (Supplementary Figure S2). When compared to mock-infected cells, the number of DEGs was lower for RV than those of IAV and IBV infection at each time point (Supplementary Figure S3). For IAV the number of DEGs increased from 147 to 2306 and for IBV from 1692 to 6788 over time. When compared to mock-infected cells, RV-infected cells gave rise to the lowest number of DEGs when compared to IAV or IBV-infected cells at all time points. In particular, there were only two DEGs for RV at 6 hpi, with very low log2 fold-change (<1.2) (Supplementary Data S3). These results suggest a delayed host response for RV when compared to influenza viruses.

Since only two DEGs were found for RV at 6 hpi, we focused on the comparison for 12 and 24 hpi (Table 1 and Supplementary Data S3). At 12 and 24 hpi, interferon-related genes and CXCL10 were highly expressed for both RV and IAV/IBV infection. IFN-λ genes (IFNL1, IFNL2, IFNL3) were the most highly expressed among the interferon genes at both 12 and 24 h post-infection for all viruses. CXCL10 was the top DEGs for all 3 viruses at 24 hpi, but also within the top 6 DEGs at 12 hpi.

**TABLE 1:** Top 20 upregulated DEGs when compared with mock-infected cells.

	IAV vs. mock		IBV vs. mock		RV vs. mock	
			
Hours post-infection	Gene name	log2 (Fold Change)	Gene name	log2 (Fold Change)	Gene name	log2 (Fold Change)
12	*IFNL2*	7.10	*IFNL1*	9.88	*IFNL2*	7.61
	*IFNL1*	7.09	*CXCL10*	9.63	*IFNL3*	7.38
	*IFNL3*	6.89	*IFNL2*	9.55	*IFNB1*	7.34
	*RSAD2*	6.76	*IFNL3*	9.36	*IFNL1*	7.14
	*CXCL10*	6.68	*IFNB1*	8.95	*AC011511.5*	6.13
	*IFNB1*	6.44	*CXCL11*	8.91	*FAP*	6.01
	*IDO1*	6.28	*IDO1*	8.59	*TULP2*	5.77
	*BATF2*	6.19	*CCL5*	8.40	*RPL7P19*	5.51
	*MX2*	5.92	*BATF2*	8.14	*ZBP1*	5.38
	*IFIT2*	5.86	*GBP5*	8.09	*AL133163.1*	5.36
	*OAS2*	5.76	*CXCL9*	8.01	*ICAM5*	5.25
	*OR52K3P*	5.58	*RSAD2*	7.89	*CXCL10*	5.12
	*IFIT3*	5.53	*GBP4*	7.70	*RSAD2*	5.02
	*AC005515.1*	5.53	*ZBP1*	7.42	*BATF2*	4.65
	*IFIT1*	5.52	*CX3CL1*	7.33	*IFIT2*	4.63
	*GBP4*	5.52	*AC005515.1*	7.24	*MX2*	4.61
	*TRIM22*	5.43	*LRP2*	7.18	*IFIT1*	4.48
	*ZBP1*	5.40	*MX2*	7.10	*OAS2*	4.46
	*ETV7*	5.40	*CD69*	6.99	*IFIT3*	4.35
	*CMPK2*	5.39	*CSF3*	6.94	*CMPK2*	4.31
24	*CXCL10*	10.32	*CXCL10*	12.08	*CXCL10*	8.90
	*CXCL11*	9.20	*CXCL11*	11.58	*IFNL3*	7.68
	*ZBP1*	9.01	*IFNL1*	10.44	*IFNL2*	7.62
	*IDO1*	8.56	*CCL5*	10.35	*CXCL11*	7.59
	*IFNL2*	8.31	*CSF3*	10.21	*ZBP1*	7.45
	*KLHDC7B*	8.26	*IFNL2*	10.07	*RSAD2*	7.21
	*IFNL3*	8.21	*IFNL3*	9.90	*MX2*	7.13
	*IFNL1*	8.11	*TNF*	8.97	*IFNL1*	6.92
	*RSAD2*	7.98	*RSAD2*	8.79	*IDO1*	6.90
	*TRIM22*	7.76	*IFNB1*	8.76	*OAS2*	6.89
	*SLC15A3*	7.69	*IL6*	8.73	*TRIM22*	6.79
	*MX2*	7.64	*ZBP1*	8.41	*CMPK2*	6.77
	*CCL5*	7.55	*IDO1*	8.33	*IFNB1*	6.71
	*BST2*	7.48	*AL021578.1*	8.28	*KLHDC7B*	6.53
	*OAS2*	7.27	*CX3CL1*	8.12	*SLC15A3*	6.47
	*GBP4*	7.21	*HSPA6*	8.04	*GBP4*	6.09
	*AC005515.1*	7.13	*CXorf49B*	7.84	*FAP*	6.01
	*CX3CL1*	7.01	*OASL*	7.83	*ETV7*	5.82
	*CMPK2*	6.94	*GBP4*	7.82	*BST2*	5.82
	*ETV7*	6.82	*CCL22*	7.71	*BATF2*	5.77

*The log2 fold changes are based on MRN normalization (cf. Supplementary Data S3).*

Next, we investigated the change over time for all 34 DEGs that originate from investigating the top 20 upregulated DEGs per virus infections vs. mock for 12 hpi. Thirty one out of 34 are also DEGs in the condition 12 hpi against 0 hpi in either mock, IAV, IBV, or RV. Eight of these 31 DEGs are shared by mock and all virus conditions, while four are shared by mock and IAV and IBV (Supplementary Figure S4). However, none of these DEGs were regulated in the same direction when comparing mock expression with virus infections for 12 hpi against 0 hpi. In fact, significant downregulation occurred for 12 of the 31 DEGs only in mock, whereas none were upregulated in mock at 12 hpi.

**FIGURE 3:**
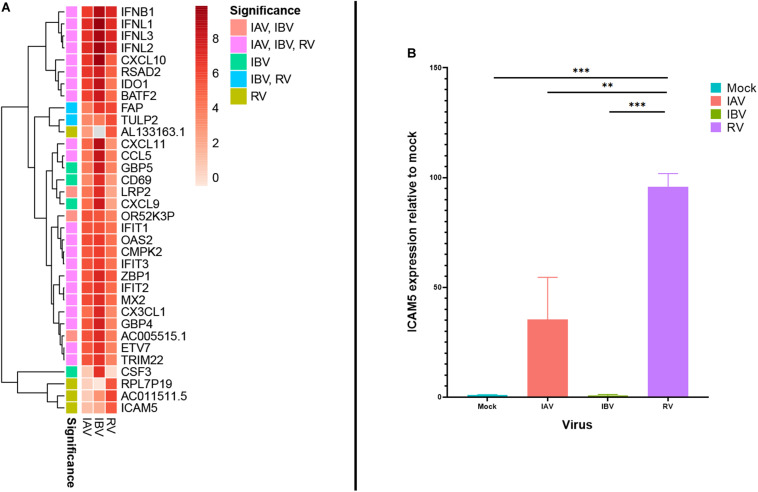
**(A)** Heatmap for top-20 differentially expressed genes with an absolute log2 fold change ≥ 2 across all virus infections with significant infection annotated. **(B)** ICAM5 expression. Monoplex ICAM5 specific real time RT-PCR was performed for IAV, IBV, and RV-infected cells. **P < 0.01; ***P <0.001.

In addition to interferons and CXCL10, several other genes were also found to be highly expressed during RV and IAV/IBV virus infection. At 12 or 24 hpi, two genes with known immune-related functions (RSAD2 and IDO1) were found to be among the top 20 DEGs for all 3 viruses (Table 1). ICAM5 was more significantly induced in cells infected with RV than those infected with IAV or IBV at 12 hpi (11th top DEG for RV; log2 fold change 5.25) (Table 1 and Figure 3A). We additionally performed ICAM5 monoplex real-time RT-PCR to confirm the expression of ICAM5 gene. ICAM-5 was expressed at a significantly higher level at 12 hpi for RV than that of IAV or IBV (Figure 3B).

#### Gene Set Enrichment Analysis (GSEA)

**FIGURE 4:**
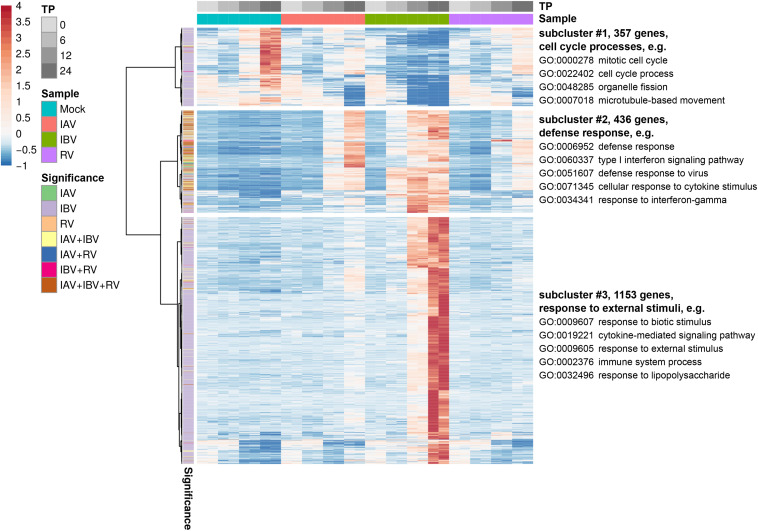
Heatmap for differentially expressed genes with an absolute log2 fold change ≥ 2 across all virus infections. Three subclusters as determined by hierarchical clustering are shown and GSEA was performed for each individual sub-cluster and the major significantly enriched pathways are indicated.

First, GSEA was performed for all three viruses at the 12 hpi time point. The majority of perturbed pathways were related to innate immune and virus response, interferons and cytokine signaling and revealed no major differentiation among different virus infections (Table 2 and Supplementary Data S4). To dissect the enriched function in the transcriptomics response further, we focused on DEGs with a log2 fold change of at least two across of all time points. We identified three major sub-clusters of the remaining 1888 transcripts across all three viruses by hierarchical clustering. GSEA was performed for each individual sub-cluster and the major significantly enriched pathways were identified, respectively (Figure 4 and Supplementary Data S5). This analysis showed that regulation of some major immune response biological processes like defense response to virus were induced by infection of all three virus types.

**TABLE 2:** Top 10 reactome pathways that enriched for IAV, IBV, or RV infection when compared with mock infection at 12 hpi using g:Profiler analysis.

	IAV			IBV			RV		
			
	Reactome pathway	FDR-adjusted *p*-value	Enrichment score	Reactome pathway	FDR-adjusted p-value	Enrichment score	Reactome pathway	FDR-adjusted *p*-value	Enrichment score
1	Interferon signaling	2.1E-27	26.69	Cell cycle	1.4E-20	19.86	Interferon signaling	4.0E-25	24.39
2	Cytokine signaling in immune system	4.1E-27	26.38	Influenza infection	1.7E-20	19.78	Interferon alpha/beta signaling	2.5E-22	21.60
3	Interferon alpha/beta signaling	1.3E-24	23.90	L13a-mediated translational silencing of ceruloplasmin expression	2.6E-20	19.58	Cytokine signaling in immune system	1.2E-17	16.92
4	Immune system	2.3E-21	20.64	Cytokine signaling in immune system	3.4E-20	19.47	Interferon gamma signaling	8.4E-13	12.08
5	Eukaryotic translation elongation	7.4E-20	19.13	Cap-dependent translation initiation	4.4E-20	19.35	Immune system	1.9E-08	7.72
6	Peptide chain elongation	7.4E-20	19.13	Eukaryotic translation initiation	4.4E-20	19.35	Interleukin-4 and Interleukin-13 signaling	6.1E-07	6.21
7	Viral mRNA translation	7.4E-20	19.13	Influenza life cycle	6.5E-20	19.18	Interleukin-10 signaling	8.5E-06	5.07
8	Nonsense mediated decay (NMD) enhanced by EJC	1.4E-19	18.85	Signaling by ROBO receptors	8.8E-20	19.06	Antiviral mechanism by IFN-stimulated genes	2.0E-05	4.70
9	Nonsense-mediated decay (NMD)	1.4E-19	18.85	Cell cycle. Mitotic	1.1E-19	18.95	FOXO-mediated transcription	9.4E-05	4.03
10	Influenza infection	1.6E-19	18.79	GTP hydrolysis and joining of the 60S ribosomal subunit	1.8E-19	18.74	Signaling by interleukins	1.5E-04	3.82

Since the number of DEGs specific to IBV infection was substantially higher (Supplementary Figure S5), we investigated enriched categories only for IBV regulated genes (Supplementary Data S6). Next to non-specific categories related to cell cycle organization, regulation and communication, we identified the enriched process “cellular response to stress,” which included DEGs like *CCL2*, *IL1B*, or *CD34* that were not differentially regulated by IAV and RV (Supplementary Data S7).

We additionally interrogated the specific role of downregulated DEGs in our data set, but could only find non-specific cell cycle, cell organization and cell communication GO biological process categories, which showcase that the cells shift to response to the infecting viruses rather than continuing cell cycle processes (Supplementary Figure S6 and Supplementary Data S8).

**FIGURE 5:**
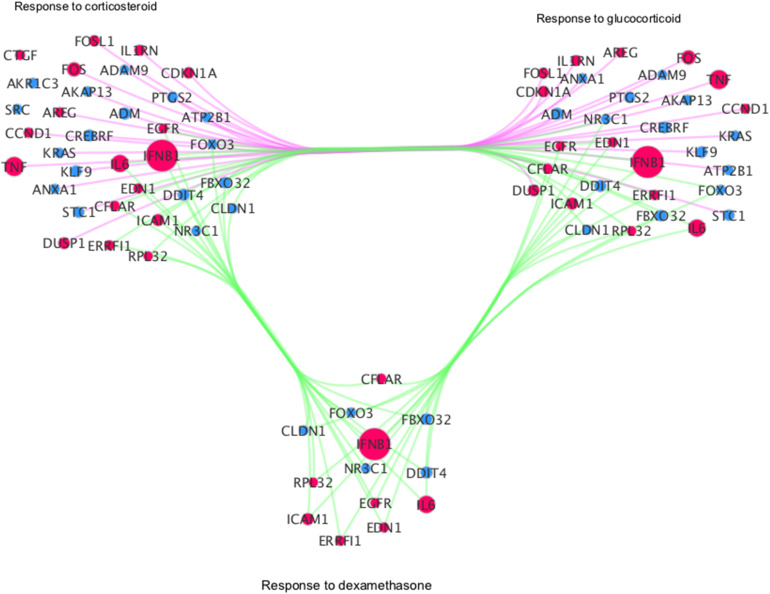
Network visualization of GSEA derived categories related to steroid biosynthesis. Red: up-regulated, Blue: down-regulated; Size: log2 fold change. Green edges: gene is shared among all categories; magenta: genes are only shared among response to corticosteroid and response to glucocorticoid categories.

In addition, we interrogated if different viruses exclusively cause differentially regulated pathways enriched by DEGs. The analysis showed that steroid-related pathways which are specifically significantly regulated in RV-infected cells relative to mock-infected subjects at 12 hpi (Supplementary Data S9). These pathways include “cellular response to mineralocorticoid stimulus” (GO:0071389), “glucocorticoid mediated signaling pathway” (GO:0043402), “regulation of glucocorticoid mediated signaling pathway” (GO:1900169), and “response to dexamethasone” (GO:0071548) (Supplementary Data S9). We identified 13 DEGs, such as EGFR or FOXO3, to be common among steroid-relevant pathways (Figure 5). Interestingly, we find upregulated IFNB1 and ICAM1 among these with proposed steroid-pathway relevancy as well. Of note, ICAM1 has been indicated to be increased by RV infection and identified to be a target for the development of therapeutic interventions for virus-induced asthma exacerbation (Papi and Johnston, 1999).

## Discussion

Both RV and influenza virus can cause severe respiratory tract infection (Hung et al., 2017). However, there are significant differences in their clinical manifestations and immune or inflammatory response (To et al., 2018). In this study, we used a transcriptomic approach to systemically compare the host response between RV and influenza virus infection in a human lower airway epithelial cell line. We have found that RV induced a more delayed and blunted host response than influenza virus infection. Interferon response and other innate immune response predominated for both RV and influenza virus infection. Interestingly ICAM5 was the only gene that was significantly upregulated for RV but not for influenza virus infection.

Our study is unique in several aspects. First, we used virus isolates that are isolated in recent years. Previous transcriptomic studies used classical strains that were isolated many years ago (Kim et al., 2015), and their results may not be representative of the contemporary viruses. Second, we infected Calu-3 cells using the same MOI to reduce the bias due to different virus titers.

The number of DEGs was much lower for RV than those of influenza virus infection. This is consistent with our previous study, which showed that the cytokine and chemokine responses were significantly lower for patients with RV infection than those with influenza virus infection (To et al., 2018). This is also consistent with the study by Zhai et al. (2015) that the overall gene expression was much stronger for patients with influenza virus infection than those with RV infection. We do note that even though the same MOI were used for all viruses a higher viral load was observed for IBV (Figure 1B), which might be partially responsible for the elevated number of DEGs expressed in Calu-3 cells when challenged with IBV. This additional set of DEGs includes interesting genes, e.g., *CCL2*, *IL1B* or *CD34* (Supplementary Data S7), which should be further investigated.

*IFNL1*, which encodes IFN-λ1, was among the top DEG for all 3 viruses at 12 h post-infection. *IFNL2* and *IFNL3*, which encode IFN-λ2 and IFN-λ3, respectively, were also induced in RV or influenza virus infected cells, but were not detectable for mock-infected cells. IFN-λ is a type III interferon, which acts via the IFN-λ receptor (Syedbasha and Egli, 2017). Through ELISA, IFN-λ was shown to be significantly expressed mainly around 24 hpi across all viruses, IBV infected samples showed significant IFN-λ expression much earlier, at 12 hpi (Figure 1D). IFN-λ1 is an important antiviral cytokine. IFN-λ has been shown to be important for the immune defense against RV. Asthmatic patients are more prone to severe RV infection, which correlated with a poorer induction of IFN-λ in airway epithelial cells and alveolar macrophages isolated from asthmatic patients than those isolated from healthy individuals (Contoli et al., 2006). In a mouse model, IFN-λ has been shown to reduce influenza virus replication, modulate immune response and protect mice from IAV infection (Davidson et al., 2016). Inhibition of *IFNL3* has been shown to increase antibody response against IAV infection (Egli et al., 2014). In a rhesus macaque model infected with influenza virus A(H5N1), IFNλ genes were also found to be highly expressed in the lung tissues (Shinya et al., 2012).

*CXCL*-*10* was highly expressed for all infections. This is compatible with our previous findings in patients, in which *CXCL10* expression was induced at high levels for both influenza virus and RV infection (To et al., 2018). This is also shown through the ELISA results, where we can see significant expression of CXCL10 at 24 hpi (Figure 1D). Our results also corroborate with the results from RNA-seq experiments on the nasopharyngeal swabs from patients with respiratory virus infection, in which *CXCL10* was one of the genes that could be used in identifying patients with respiratory virus infection (Landry and Foxman, 2018).

At 12 or 24 h, most of common genes among the top 20 DEGs were interferons or interferon-inducible genes *(IFNL1, IFNB1, RSAD2, IDO1)* or chemokines *(CXCL10, CXCL11)*. *RSAD2* and *IDO1* are mainly triggered by interferons as part of the concerted counteraction against viral infection (Duschene and Broderick, 2012; Gaelings et al., 2017). Interestingly, *IRF7*, though differentially regulated (Supplementary Data S3), was not among the top differentially regulated genes, although it possesses a pivotal role in virus triggered IFN type I induction. *GBP4* is among our top regulated genes across all viruses and was reported to interact with *IRF7* in a negative manner (Hu et al., 2011). *BATF2* is a transcription factor that controls the differentiation of dendritic cells. *BATF2* has been identified to be one of the biomarkers that can predict the progression of active tuberculosis for individuals who have close contact with tuberculosis patients (Roe et al., 2019). Batf2-/- mice had more severe *Trypansoma* infection (Kitada et al., 2017).

A previous study by Kim et al. (2015) compared the transcriptomic profiles between RV and influenza virus in a human bronchial cell line BEAS-2B. Similar to our study, they have demonstrated that IFNB1, CXCL10, CXCL11, CCL5 was upregulated in both IAV and RV infection. However, while our current study in Calu-3 cells showed significant induction of IFNλ genes, the study by Kim et al. in BEAS-2B cell line did not show induction of these genes after infection. One major limitation associated with BEAS-2B cell line is that there is a high basal production of interferon-stimulated genes, such as IRF7, ISG15, MX1, STING, which may affect the response of other host genes and also lead to Influenza A virus resistance (Seng et al., 2014).

ICAM5 was strongly expressed during RV infection, which was verified by monoplex RT-qPCR. Although ICAM5 is unlikely to be a single factor to account for the difference between RV and influenza virus, the fact that ICAM5 is much more highly expressed in RV infected cells than both influenza A and influenza B virus strongly suggests that ICAM5 may play a major role in the pathogenesis of RV. ICAM5 is a known receptor for enterovirus D68, which also belongs to the *Picornaviridae* family (Wei et al., 2016). Nonetheless. the significance of ICAM5 on RV infection deserves further studies.

Our network pathway enrichment analysis showed that steroid-related pathways are enriched. RV is more likely to be associated with acute exacerbation of asthma than influenza virus infection (To et al., 2019). A previous study has shown that RV infection leads to steroid-resistance in airway epithelium (Papi et al., 2013). Therefore, our transcriptomic analysis reveals that the difference in steroid pathways may be associated with the clinical manifestations.

Though there are studies which have analyzed the transcriptome of influenza virus or RV infection, most did not compare these viruses together. In a study with experimental human infections, comparison of blood mRNA expression showed that *SOCS1* gene were uniquely expressed for RV infection when compared with IAV and RSV (Zaas et al., 2009). However, in our study, *SOCS1* was upregulated for all 3 viruses, with higher levels among influenza virus infected cells.

Our study has demonstrated that there are some important differences like *ICAM5* expression which may explain the clinical findings of these viral infections. To generalize on our findings future work should include additional subtypes from different viruses on top of the strains we used in the present study. Our investigation revealed a number of genes that are similarly expressed upon infection with any of the studied viruses. These genes may yield broad spectrum antivirals for the treatment of influenza virus and RV infection.

## Data Availability Statement

The datasets generated for this study can be found in the NCBI Sequence Read Archive (accession number PRJNA609228).

## Author Contributions

TD and KT contributed to the conception and design of the work, data acquisition, data analysis, interpretation of the data, and drafting and revising the manuscript critically for intellectual content. M-LY and K-YY contributed to the conception and design of the work, data analysis, interpretation of the data, and revising the manuscript critically for intellectual content. GP contributed to the design of the work, data analysis, interpretation of the data, and revising the manuscript critically for intellectual content. SS, MM, W-LW, AN, CY, AL, TW, and K-HC contributed to the data analysis and interpretation of data, and revising the manuscript critically for intellectual content. All authors contributed to the article and approved the submitted version.

## Conflict of Interest

The authors declare that the research was conducted in the absence of any commercial or financial relationships that could be construed as a potential conflict of interest.
