# Corrigendum: Comparative Transcriptomic Analysis of Rhinovirus and Influenza Virus Infection

**DOI:** 10.3389/fmicb.2020.602854

**Published:** 2020-10-29

**Authors:** Thrimendra Kaushika Dissanayake, Sascha Schäuble, Mohammad Hassan Mirhakkak, Wai-Lan Wu, Anthony Chin-Ki Ng, Cyril C. Y. Yip, Albert García López, Thomas Wolf, Man-Lung Yeung, Kwok-Hung Chan, Kwok-Yung Yuen, Gianni Panagiotou, Kelvin Kai-Wang To

**Affiliations:** ^1^Department of Microbiology, Li Ka Shing Faculty of Medicine, The University of Hong Kong, Hong Kong, China; ^2^Systems Biology and Bioinformatics Unit, Leibniz Institute for Natural Product Research and Infection Biology – Hans Knöll Institute, Jena, Germany; ^3^State Key Laboratory for Emerging Infectious Diseases, Li Ka Shing Faculty of Medicine, The University of Hong Kong, Hong Kong, China; ^4^Department of Clinical Microbiology and Infection Control, The University of Hong Kong, Hong Kong, China; ^5^Carol Yu Centre for Infection, The University of Hong Kong, Hong Kong, China; ^6^Systems Biology and Bioinformatics Group, School of Biological Sciences, Faculty of Sciences, The University of Hong Kong, Hong Kong, China

**Keywords:** influenza, rhinovirus, transcriptomics analysis, ICAM5, interferons, cytokines

An author name was incorrectly spelled as Mohammad Mirhakkak. The correct spelling is Mohammad Hassan Mirhakkak.

Thomas Wolf was not included as an author in the published article. The corrected Author Contributions Statement appears below.

TD and KT contributed to the conception and design of the work, data acquisition, data analysis, interpretation of the data, and drafting and revising the manuscript critically for intellectual content. M-LY and K-YY contributed to the conception and design of the work, data analysis, interpretation of the data, and revising the manuscript critically for intellectual content. GP contributed to the design of the work, data analysis, interpretation of the data, and revising the manuscript critically for intellectual content. SS, MM, W-LW, AN, CY, AL, TW, and K-HC contributed to the data analysis and interpretation of data, and revising the manuscript critically for intellectual content. All authors contributed to the article and approved the submitted version.

The authors apologize for this error and state that this does not change the scientific conclusions of the article in any way. The original article has been updated.

