# Supplementary material for: Comparative Transcriptomic Analysis of Rhinovirus and Influenza Virus Infection
Source: Front Microbiol. 2020 Jul 21;11:1580. doi: 10.3389/fmicb.2020.01580 (PMC7396524; doi:10.3389/fmicb.2020.01580)
Supplement: Supplementary file 1 [file Data_Sheet_1.PDF]

General Statistics

Sample Name	% Dropped	% rRNA	% Dups	% GC	M Seqs	% Aligned	M Aligned	M Reads Mapped	% Assigned	M Assigned
**10Nil6_1**	0.7%	6.4%	56.8%	50%	25.4	93.2%	23.3	47.4	90.6%	21.5
**11FluA6_1**	0.7%	4.1%	57.2%	49%	26.9	92.9%	24.6	50	91.0%	22.8
**12FluA6_1**	0.7%	3.4%	60.3%	49%	31.9	93.2%	29.2	59.5	91.0%	27
**13FluB6_1**	0.6%	4.8%	61.8%	48%	27.5	84.2%	22.8	46.3	91.1%	21.1
**14FluB6_1**	0.7%	1.7%	61.8%	48%	28	83.1%	22.9	46.6	91.1%	21.2
**15Rhino6_1**	0.7%	5.4%	59.7%	49%	29.8	93.5%	27.4	55.7	90.8%	25.3
**16Rhino6_1**	0.8%	4.0%	58.9%	49%	29.8	93.5%	27.4	55.7	90.9%	25.3
**17Nil12_1**	0.7%	5.6%	59.5%	49%	29.4	93.9%	27.1	55.2	91.2%	25.2
**18Nil12_1**	0.8%	4.5%	56.3%	49%	23.9	94.1%	22.1	44.9	91.1%	20.5
**19FluA12_1**	0.8%	4.0%	57.3%	49%	25.6	91.6%	23.1	46.9	91.1%	21.4
**1Nil0_1**	0.7%	6.5%	57.6%	50%	27.1	94.1%	25	51	90.6%	23.1
**20FluA12_1**	0.8%	5.2%	59.9%	49%	29.5	91.5%	26.5	53.9	91.3%	24.6
**21FluB12_1**	0.7%	5.5%	71.3%	46%	28.8	55.9%	15.8	32.2	90.5%	14.6
**22FluB12_1**	0.7%	3.7%	71.7%	46%	27.6	54.2%	14.7	29.9	90.3%	13.5
**23Rhino12_1**	0.7%	2.4%	57.9%	49%	28.2	92.2%	25.5	51.9	91.0%	23.6
**24Rhino12_1**	0.7%	5.0%	58.3%	49%	28	92.3%	25.4	51.7	91.1%	23.5
**25Nil24_1**	0.7%	5.7%	56.8%	49%	25.5	93.3%	23.4	47.5	90.4%	21.5
**26Nil24_1**	0.8%	3.6%	57.8%	49%	26.3	93.3%	24.1	49	90.5%	22.2
**27FluA24_1**	0.7%	8.0%	57.2%	49%	25.1	88.8%	21.9	44.5	90.0%	20
**28FluA24_1**	0.7%	6.6%	57.0%	49%	25.4	89.3%	22.4	45.4	90.1%	20.5
**29FluB24_1**	0.6%	3.9%	75.2%	44%	29.1	40.9%	11.7	23.8	85.8%	10.2
**2Nil0_1**	0.6%	4.9%	57.4%	49%	26.1	94.2%	24.1	49.1	90.6%	22.2
**30FluB24_1**	0.7%	5.2%	76.1%	44%	26	37.7%	9.6	19.6	86.7%	8.5
**31Rhino24_1**	0.7%	6.4%	58.2%	49%	29.5	93.0%	27	54.8	91.1%	25
**32Rhino24_1**	1.0%	6.2%	58.2%	49%	27.6	93.0%	25.3	51.4	91.2%	23.4
**3FluA0_1**	0.6%	6.3%	57.8%	49%	26.5	94.0%	24.5	49.8	90.6%	22.5
**4FluA0_1**	0.6%	6.9%	57.0%	49%	25.8	94.0%	23.8	48.5	90.6%	22
**5FluB0_1**	0.6%	2.6%	59.2%	49%	29.3	94.1%	27.1	55.1	90.7%	25
**6FluB0_1**	0.6%	6.3%	57.7%	49%	27.1	94.3%	25.1	51.1	90.7%	23.2
**7Rhino0_1**	0.7%	5.8%	57.4%	49%	27.4	93.8%	25.2	51.4	90.4%	23.2
**8Rhino0_1**	0.7%	6.4%	58.5%	49%	27.9	93.5%	25.7	52.2	90.5%	23.6
**9Nil6_1**	1.1%	2.7%	60.4%	49%	30.6	93.1%	28	57	90.8%	25.9

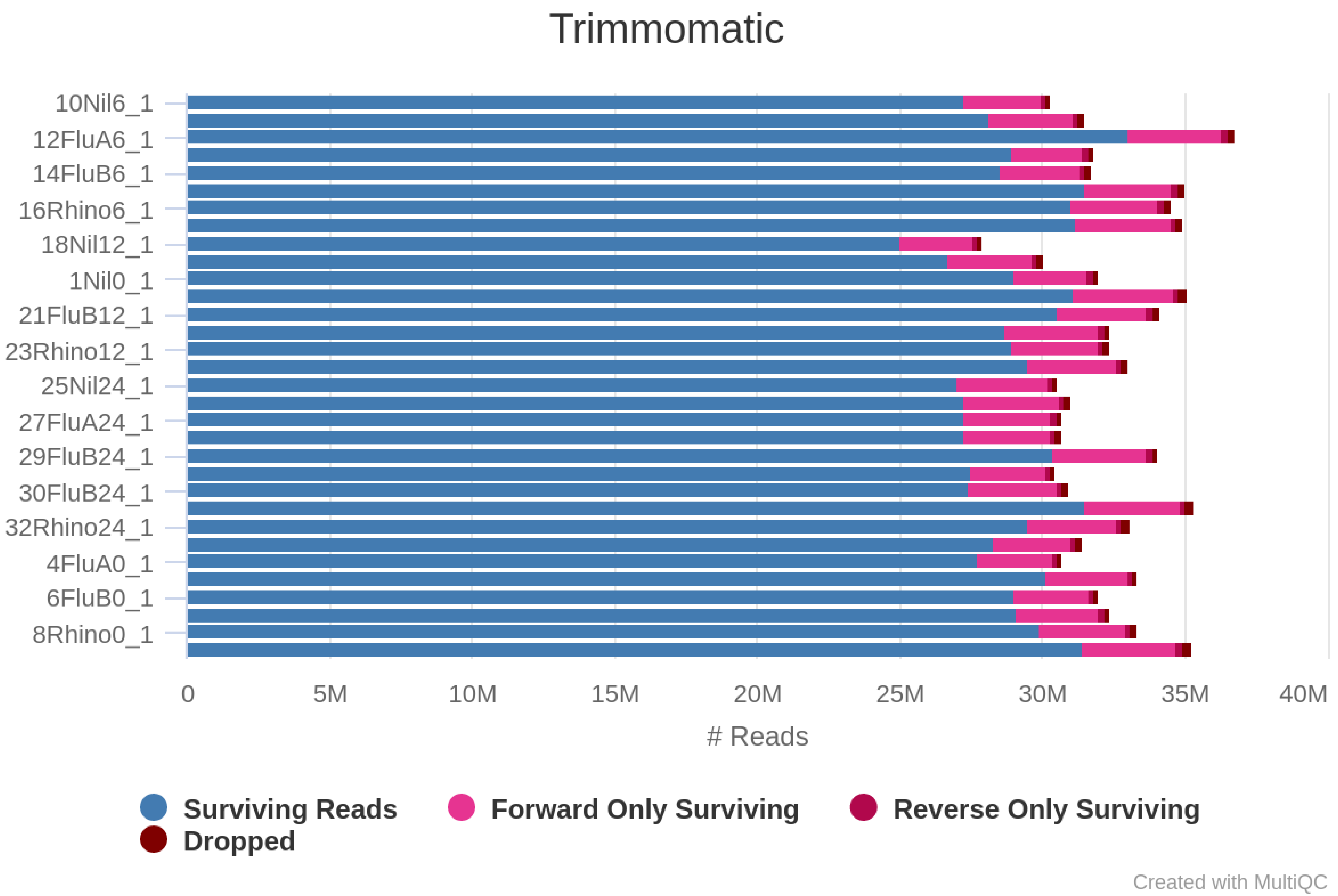
Trimmomatic
10Nil6_1
12FluA6_1
14FluB6_1
16Rhino6_1
18Nil12_1
1Nil0_1
21FluB12_1
23Rhino12_1
25Nil24_1
27FluA24_1
29FluB24_1
30FluB24_1
32Rhino24_1
4FluA0_1
6FluB0_1
8Rhino0_1
0
5M
10M
15M
20M
25M
30M
35M
40M
Reads
Surviving Reads
Forward Only Surviving
Reverse Only Surviving
Dropped
Created with MultiQC

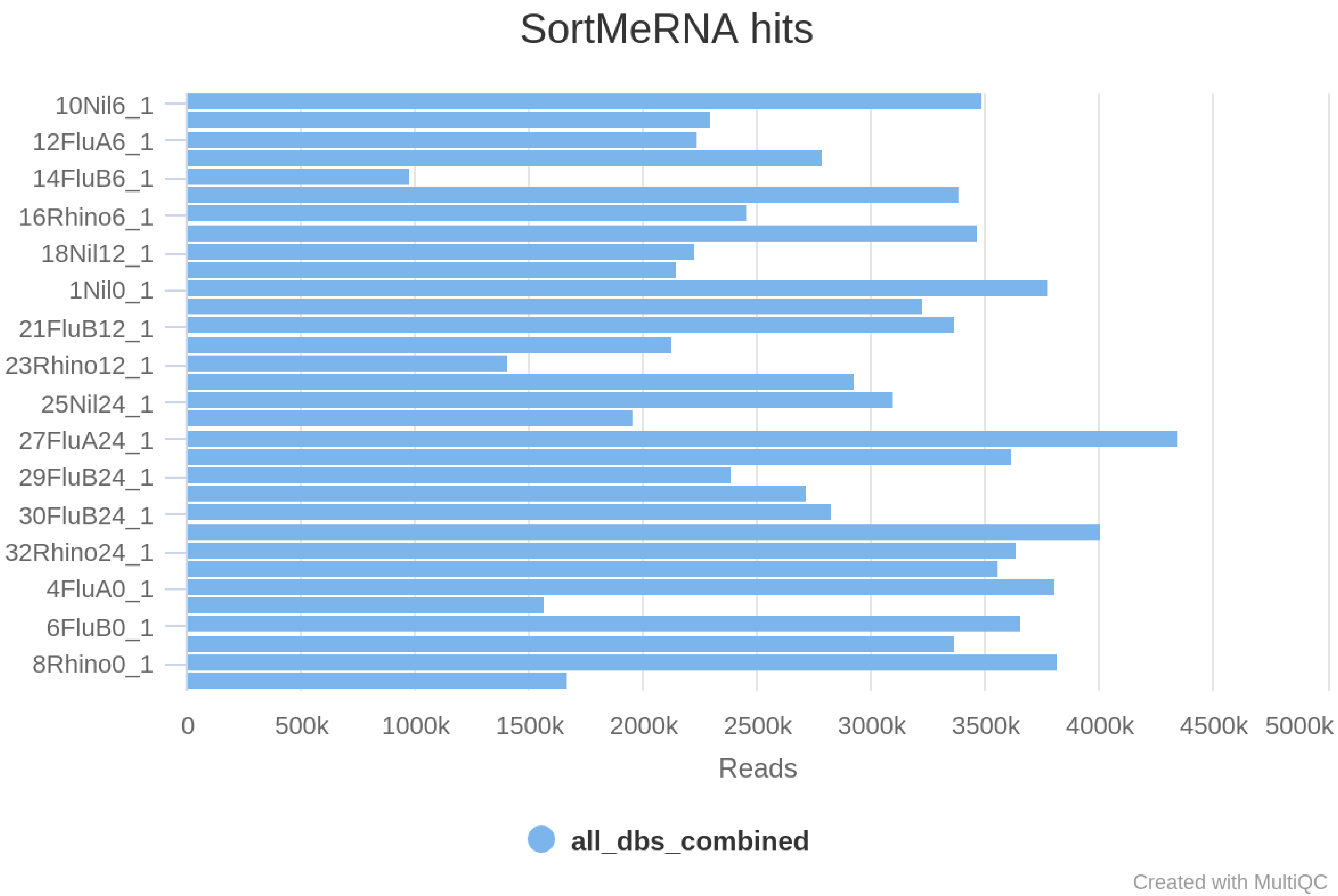
SortMeRNA hits
10Nil6_1
12FluA6_1
14FluB6_1
16Rhino6_1
18Nil12_1
1Nil0_1
21FluB12_1
23Rhino12_1
25Nil24_1
27FluA24_1
29FluB24_1
30FluB24_1
32Rhino24_1
4FluA0_1
6FluB0_1
8Rhino0_1
0
500k
1000k
1500k
2000k
2500k
3000k
3500k
4000k
4500k
5000k
Reads
all_dbs_combined
Created with MultiQC

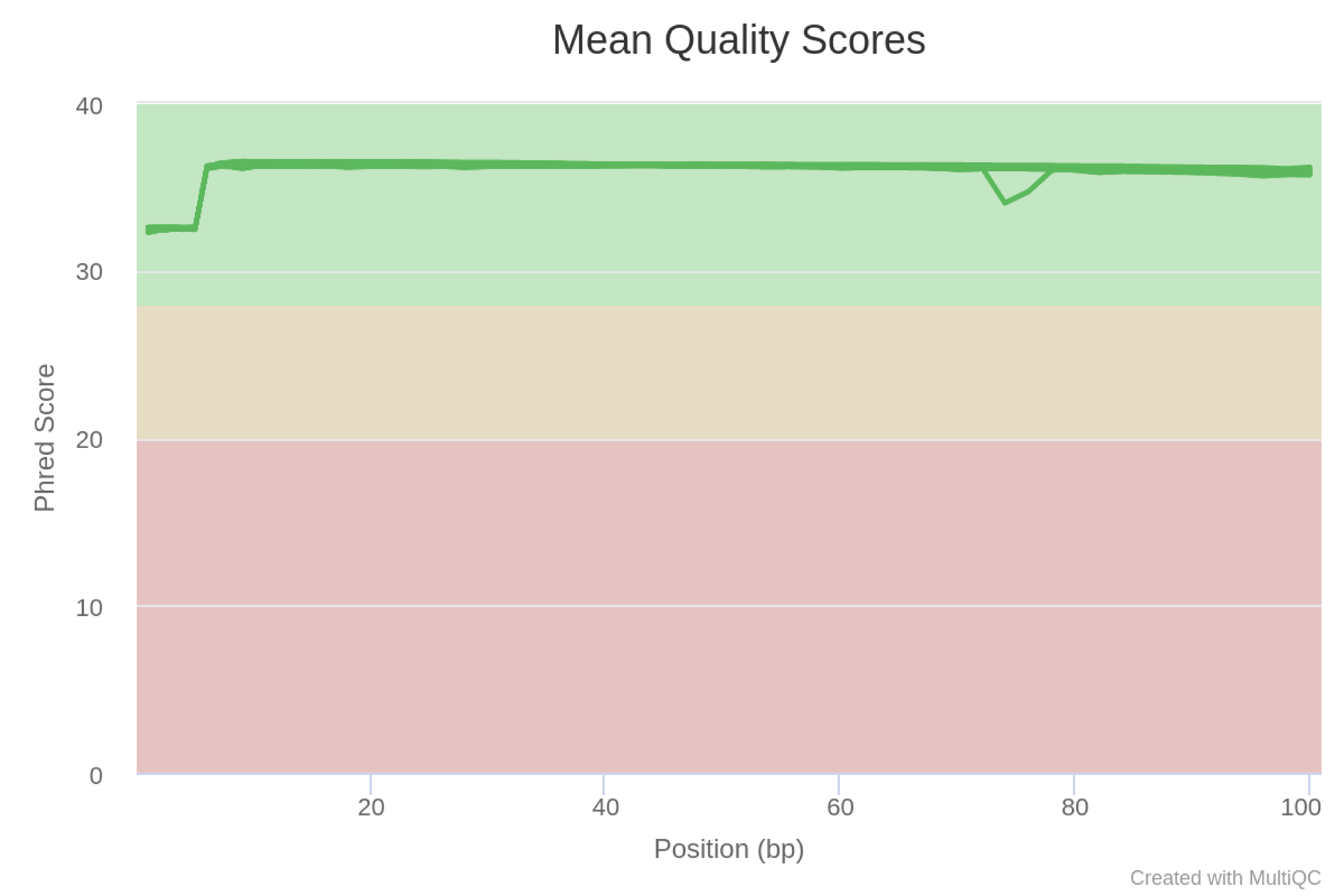

Mean Quality Scores
40
30
20
10
0
Phred Score
20
40
60
80
100
Position (bp)
Created with MultiQC

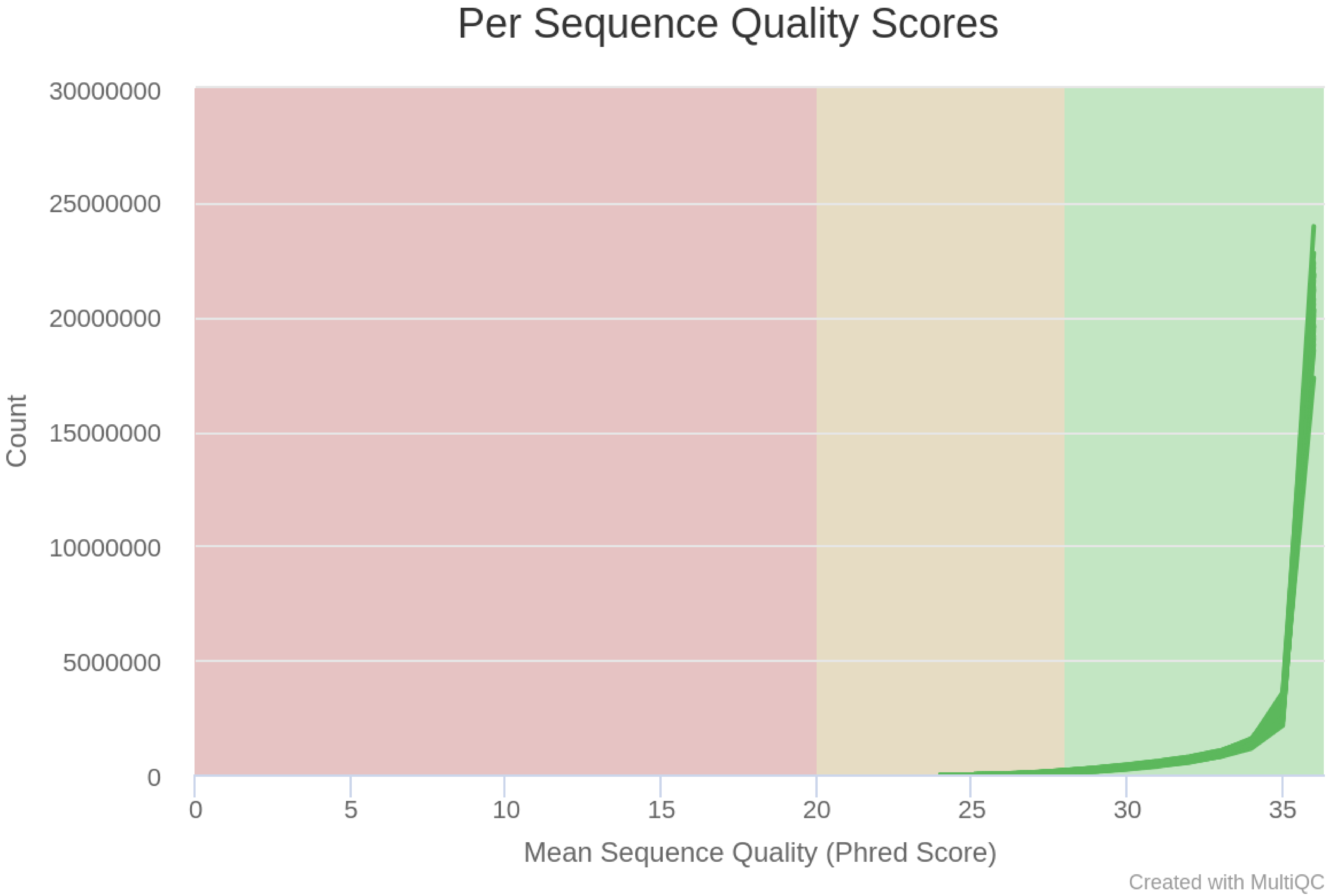

Per Sequence Quality Scores
30000000
25000000
20000000
15000000
10000000
5000000
0
Count
0
5
10
15
20
25
30
35
Mean Sequence Quality (Phred Score)
Created with MultiQC

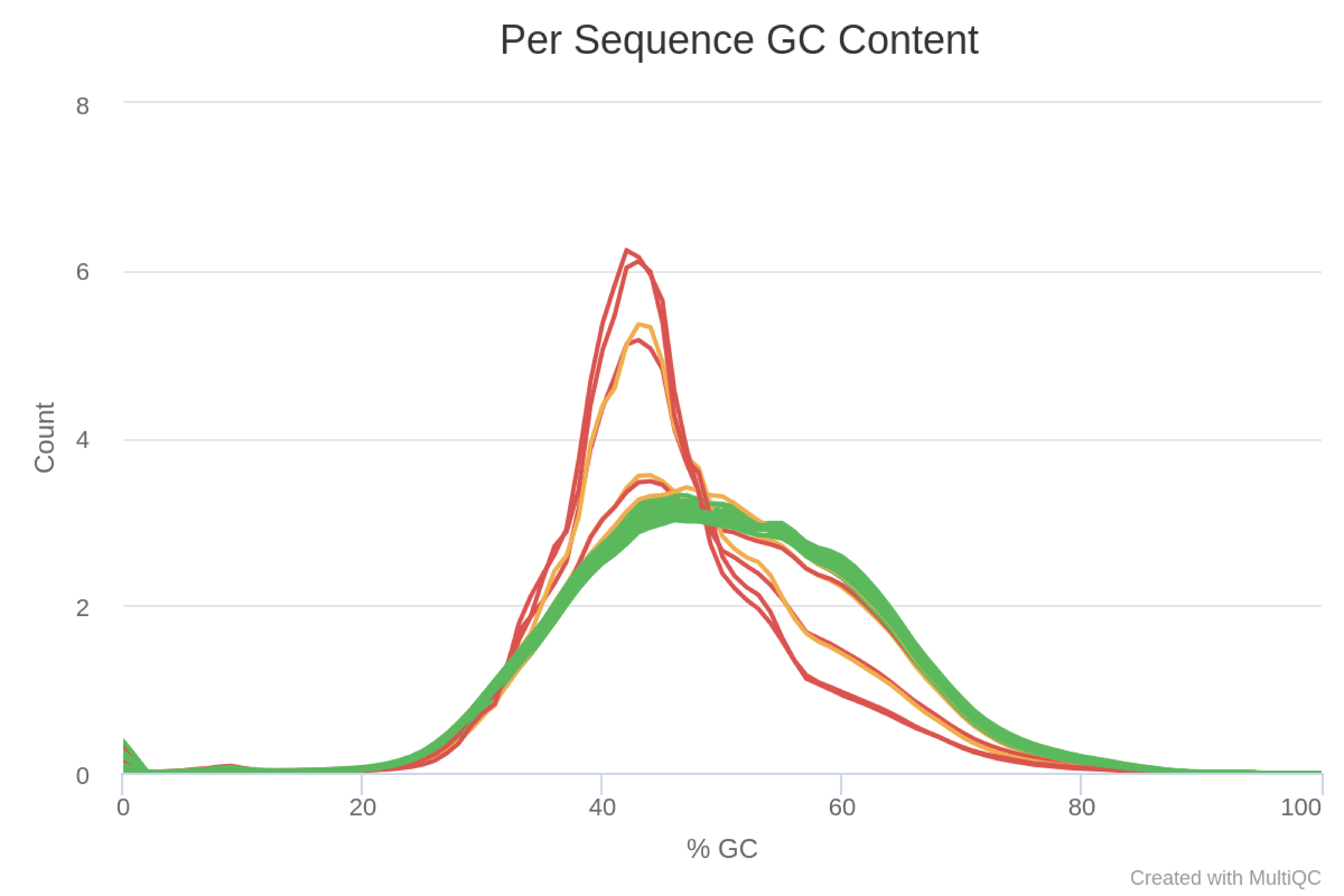

Per Sequence GC Content
Count
8
6
4
2
0
0
20
40
60
80
100
% GC
Created with MultiQC

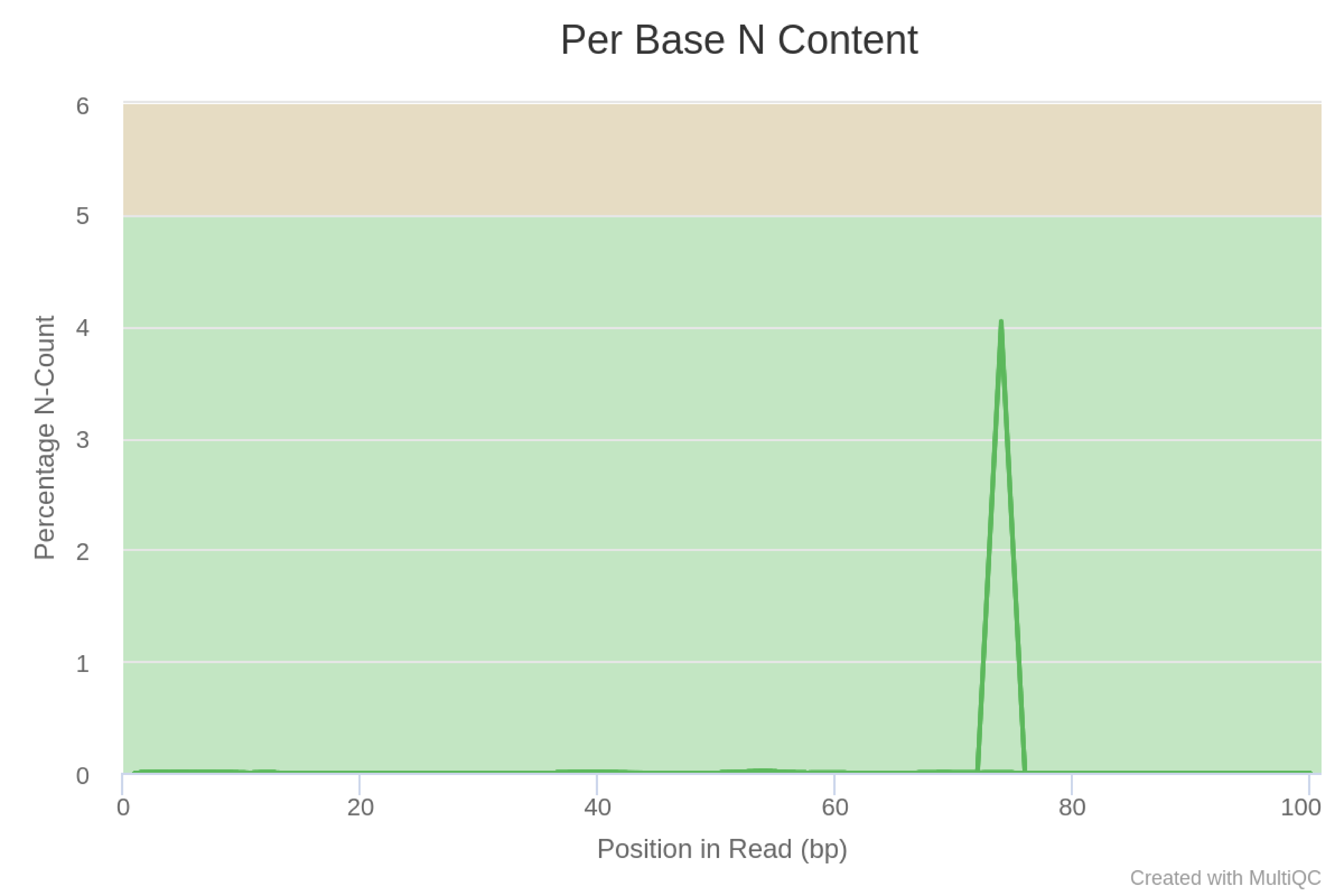

Per Base N Content
Percentage N-Count
0
1
2
3
4
5
6
0
20
40
60
80
100
Position in Read (bp)
Created with MultiQC

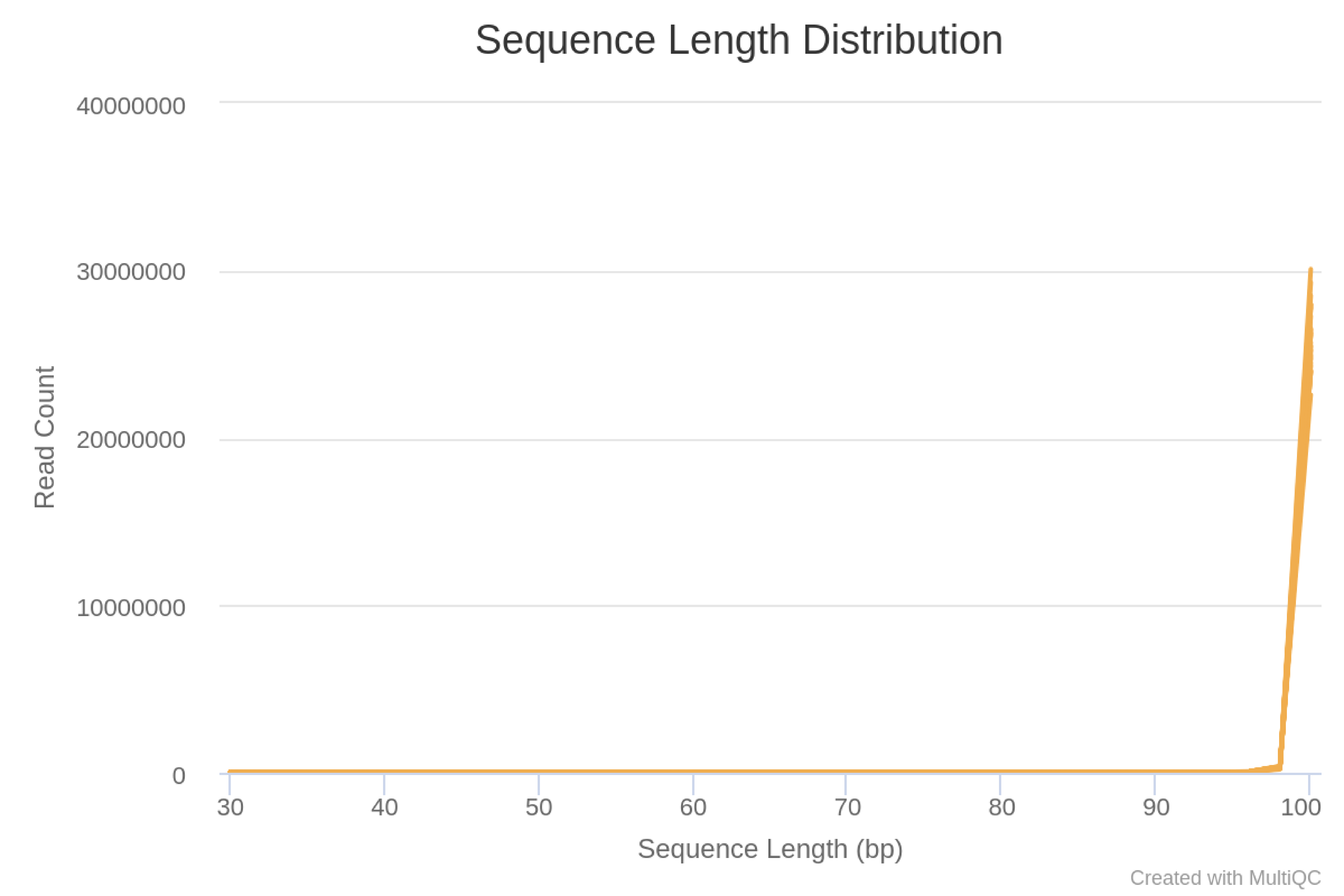
Sequence Length Distribution
Read Count
40000000
30000000
20000000
10000000
0
30
40
50
60
70
80
90
100
Sequence Length (bp)
Created with MultiQC

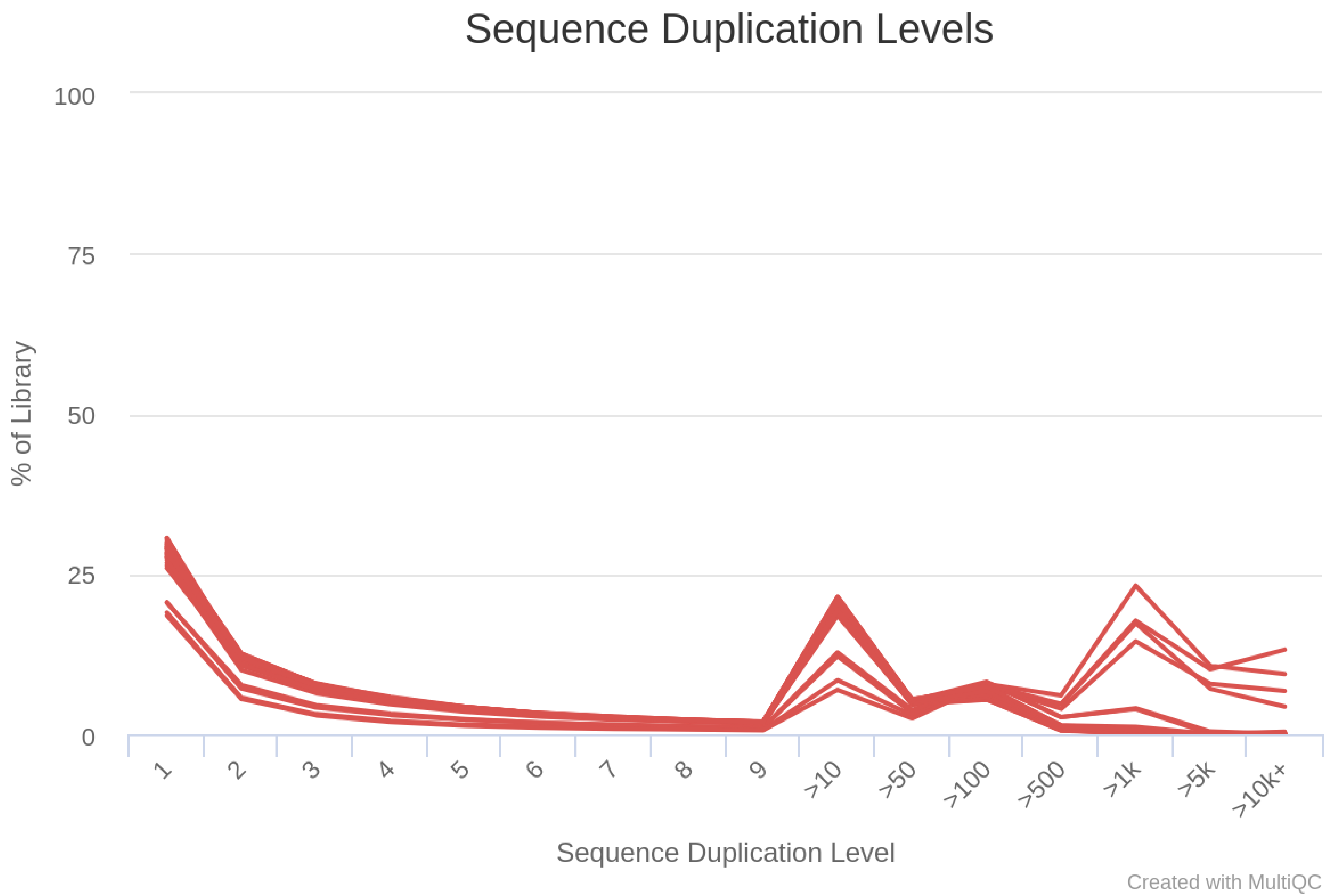
Sequence Duplication Levels
100
75
50
25
0
% of Library
1
2
3
4
5
6
7
8
9
>10
>50
>100
>500
>1k
>5k
>10k+
Sequence Duplication Level
Created with MultiQC

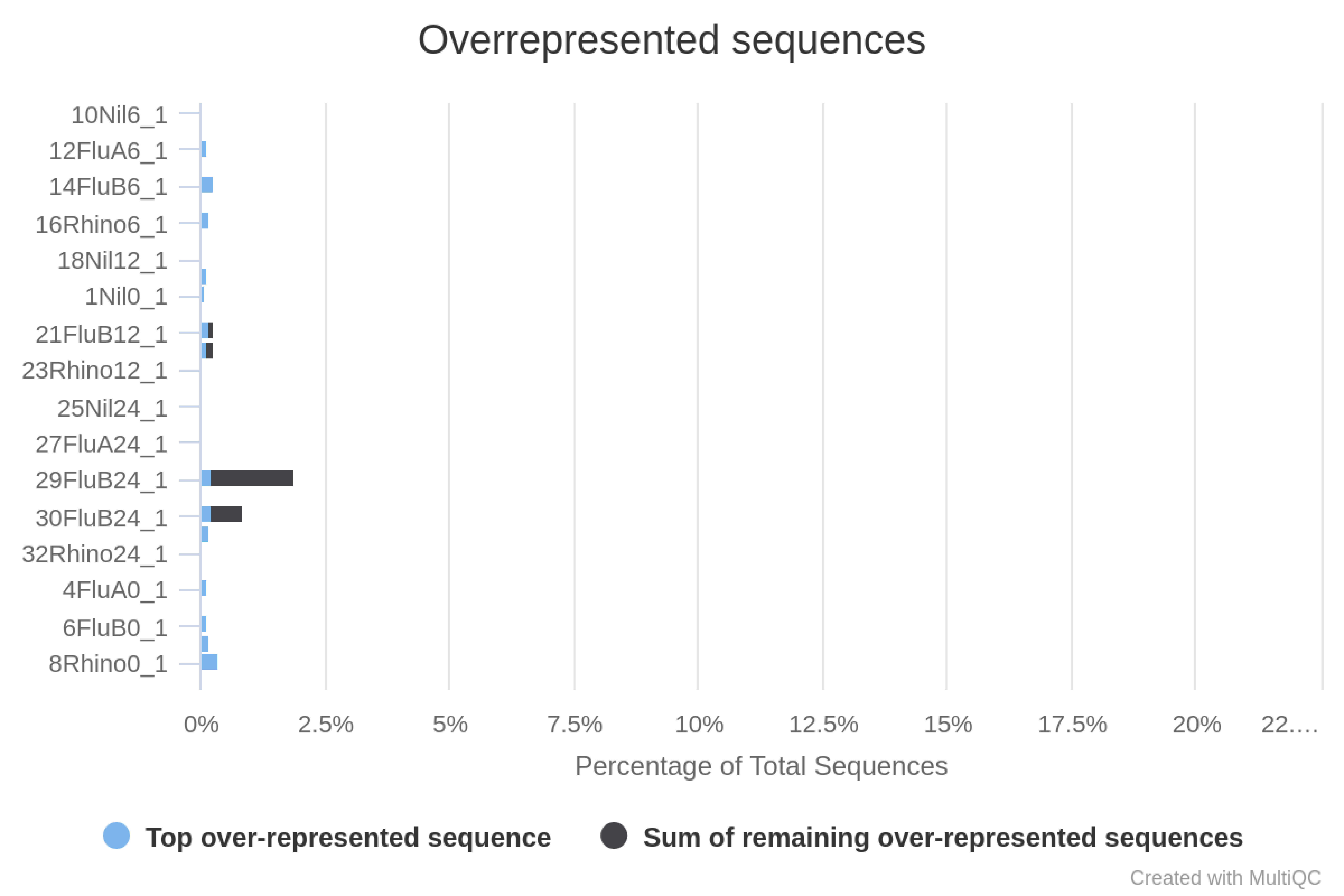

Overrepresented sequences
10Nil6_1
12FluA6_1
14FluB6_1
16Rhino6_1
18Nil12_1
1Nil0_1
21FluB12_1
23Rhino12_1
25Nil24_1
27FluA24_1
29FluB24_1
30FluB24_1
32Rhino24_1
4FluA0_1
6FluB0_1
8Rhino0_1
0%
2.5%
5%
7.5%
10%
12.5%
15%
17.5%
20%
22.…
Percentage of Total Sequences
Top over-represented sequence
Sum of remaining over-represented sequences
Created with MultiQC

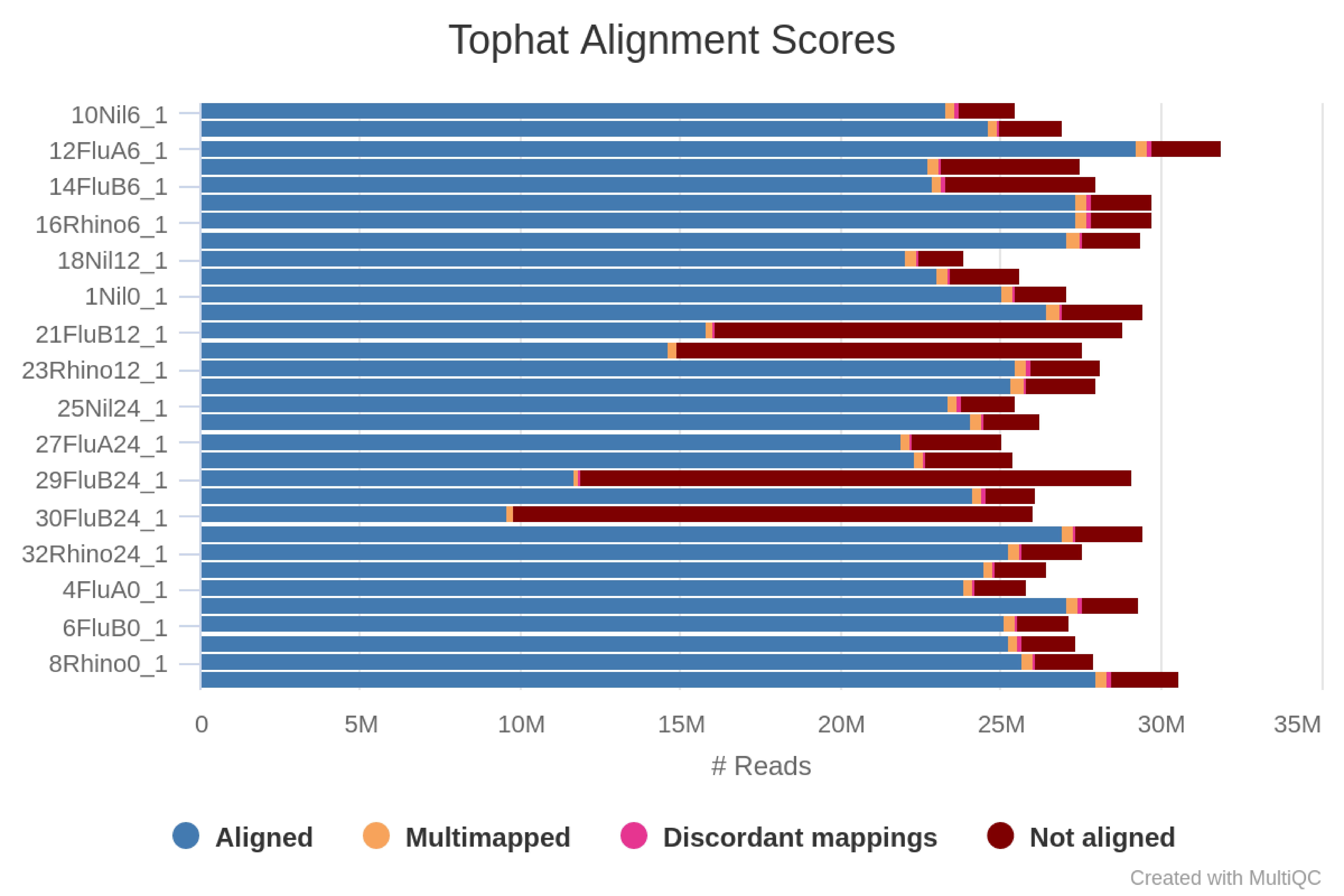

Tophat Alignment Scores
10Nil6_1
12FluA6_1
14FluB6_1
16Rhino6_1
18Nil12_1
1Nil0_1
21FluB12_1
23Rhino12_1
25Nil24_1
27FluA24_1
29FluB24_1
30FluB24_1
32Rhino24_1
4FluA0_1
6FluB0_1
8Rhino0_1
0
5M
10M
15M
20M
25M
30M
35M
Reads
Aligned
Multimapped
Discordant mappings
Not aligned
Created with MultiQC

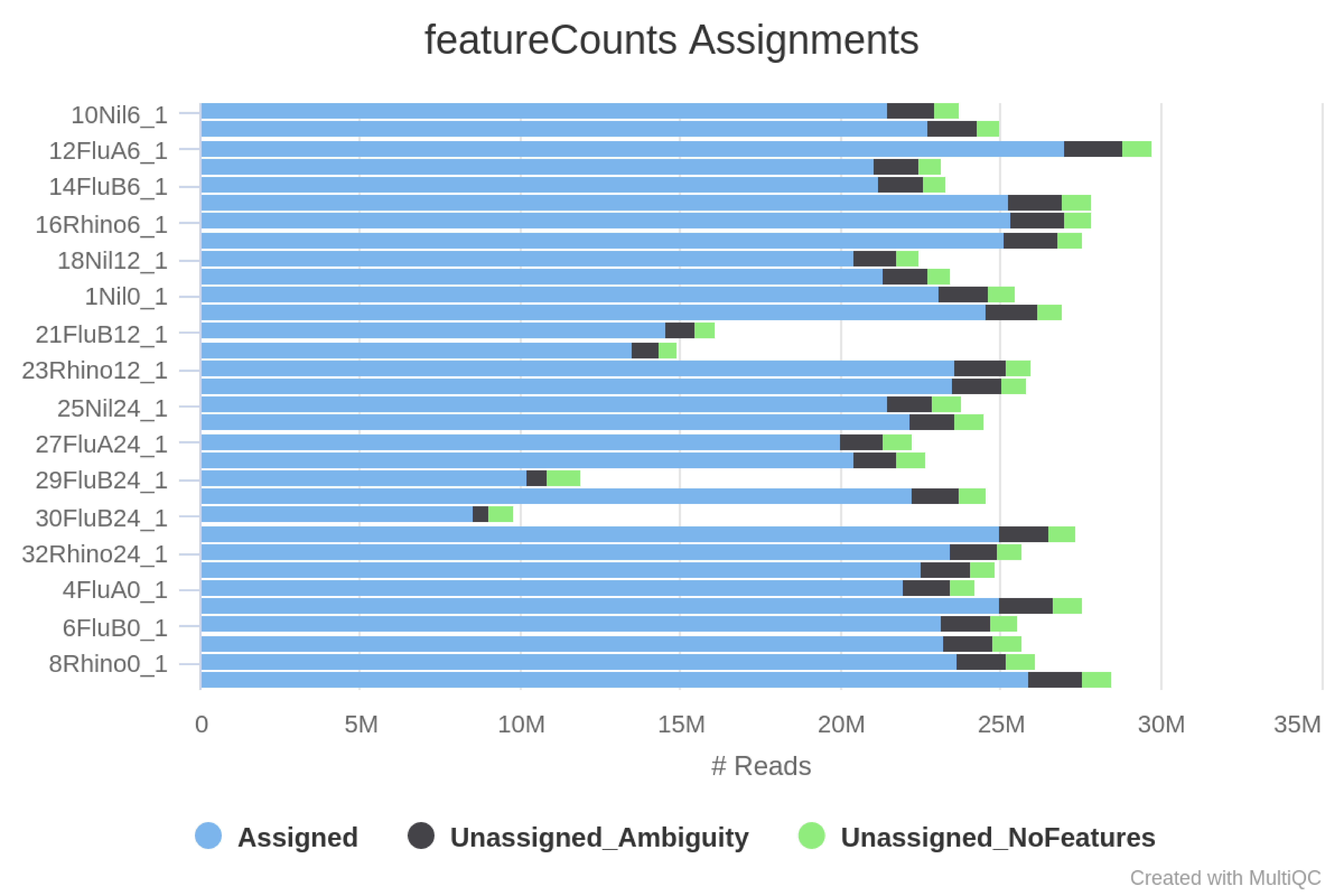
featureCounts Assignments
10Nil6_1
12FluA6_1
14FluB6_1
16Rhino6_1
18Nil12_1
1Nil0_1
21FluB12_1
23Rhino12_1
25Nil24_1
27FluA24_1
29FluB24_1
30FluB24_1
32Rhino24_1
4FluA0_1
6FluB0_1
8Rhino0_1
0
5M
10M
15M
20M
25M
30M
35M
Reads
Assigned
Unassigned_Ambiguity
Unassigned_NoFeatures
Created with MultiQC
