# Supplementary material for: Comparative Transcriptomic Analysis of Rhinovirus and Influenza Virus Infection
Source: Front Microbiol. 2020 Jul 21;11:1580. doi: 10.3389/fmicb.2020.01580 (PMC7396524; doi:10.3389/fmicb.2020.01580)
Supplement: Supplementary file 10 [file Image_1.PDF]

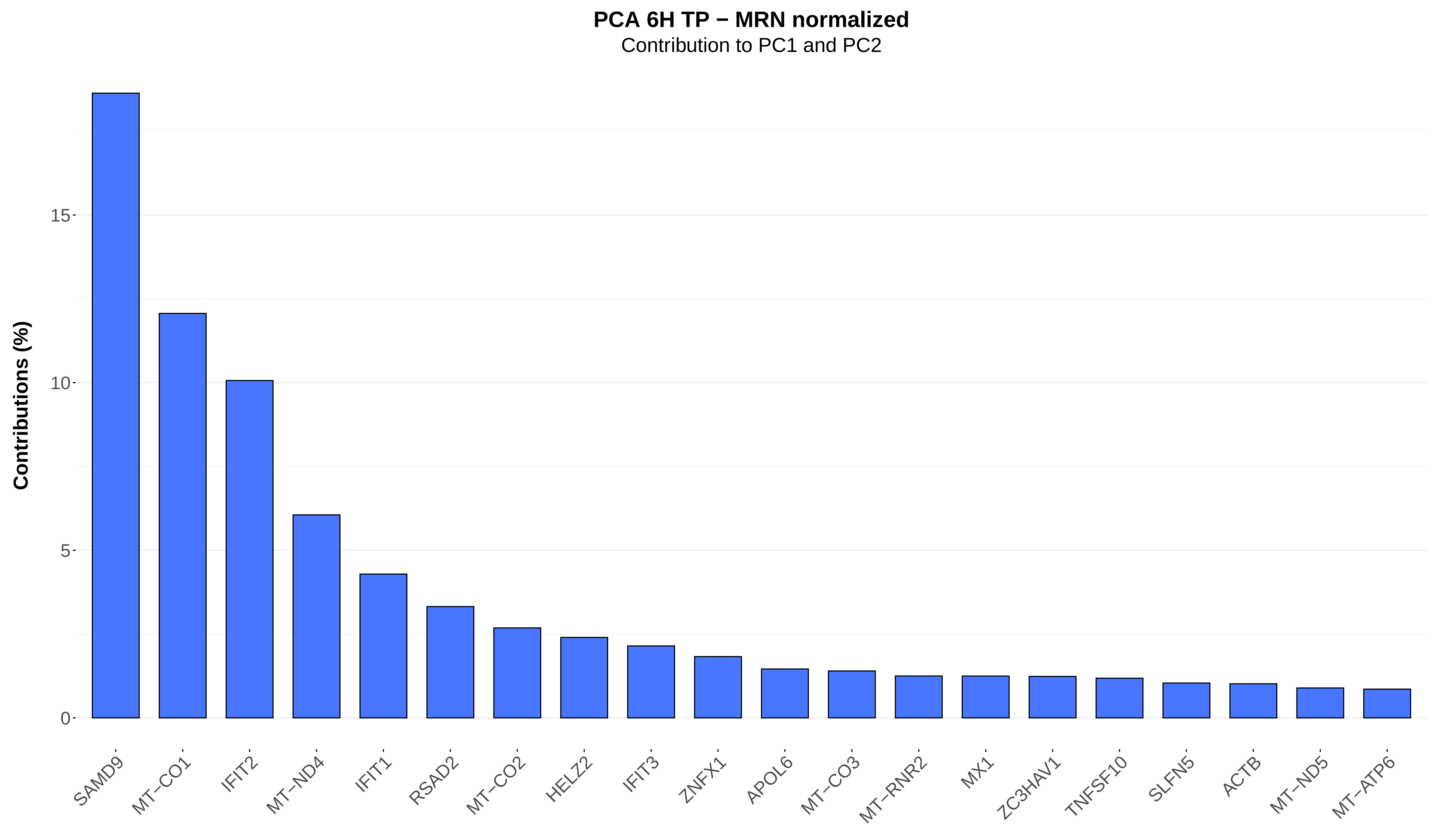

PCA 6H TP − MRN normalized
Contribution to PC1 and PC2
Contributions (%)
0
5
10
15
SAMD9
MT−CO1
IFIT2
MT−ND4
IFIT1
RSAD2
MT−CO2
HELZ2
IFIT3
ZNFX1
APOL6
MT−CO3
MT−RNR2
MX1
ZC3HAV1
TNFSF10
SLFN5
ACTB
MT−ND5
MT−ATP6

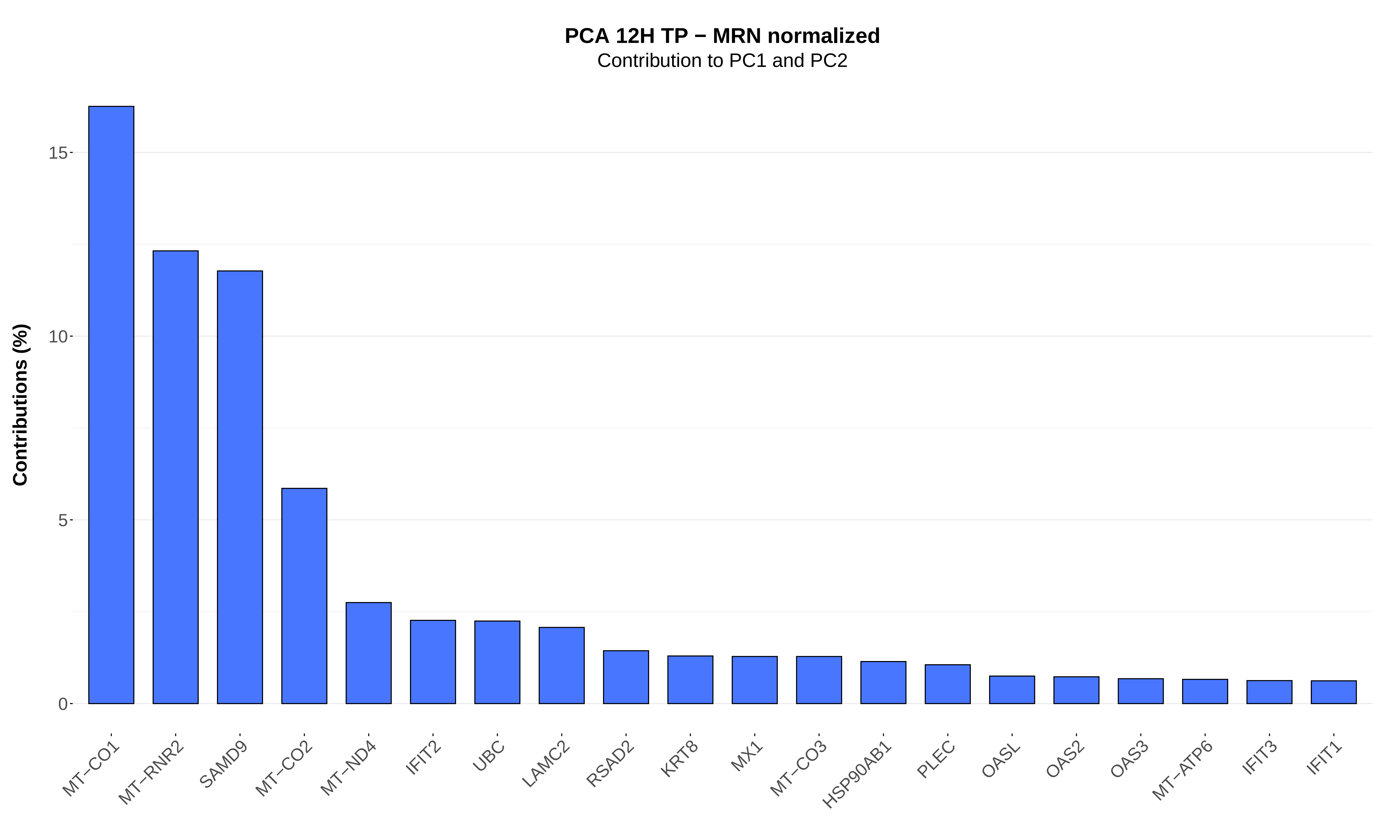
PCA 12H TP − MRN normalized
Contribution to PC1 and PC2
Contributions (%)
15
10
5
0
MT−CO1
MT−RNR2
SAMD9
MT−CO2
MT−ND4
IFIT2
UBC
LAMC2
RSAD2
KRT8
MX1
MT−CO3
HSP90AB1
PLEC
OASL
OAS2
OAS3
MT−ATP6
IFIT3
IFIT1

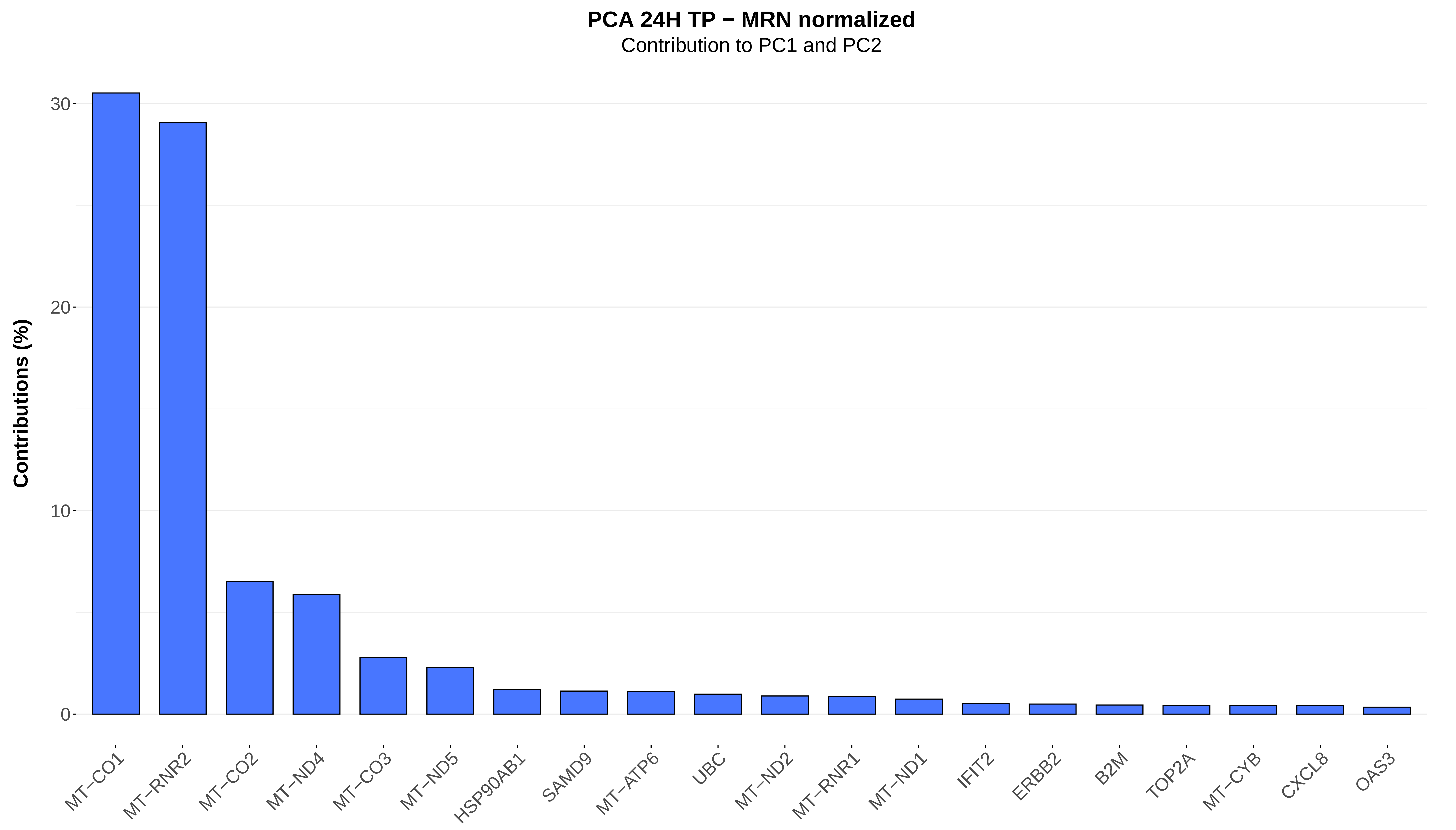

PCA 24H TP − MRN normalized
Contribution to PC1 and PC2
Contributions (%)
0
10
20
30
MT−CO1
MT−RNR2
MT−CO2
MT−ND4
MT−CO3
MT−ND5
HSP90AB1
SAMD9
MT−ATP6
UBC
MT−ND2
MT−RNR1
MT−ND1
IFIT2
ERBB2
B2M
TOP2A
MT−CYB
CXCL8
OAS3
