# Supplementary figures and images for: Comparative Transcriptomic Analysis of Rhinovirus and Influenza Virus Infection
Source: Front Microbiol. 2020 Jul 21;11:1580. doi: 10.3389/fmicb.2020.01580 (PMC7396524; doi:10.3389/fmicb.2020.01580)

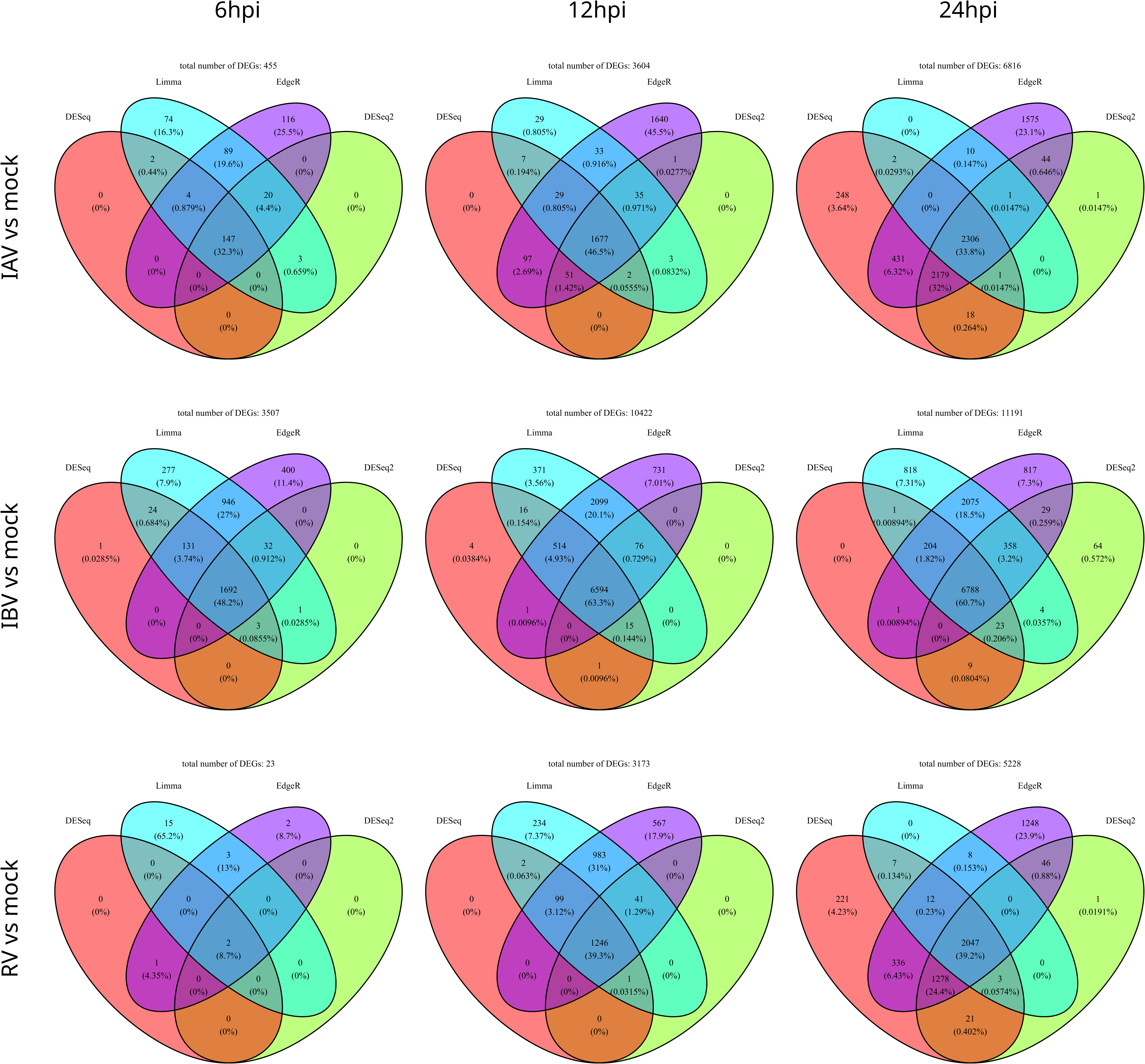

Supplement: Supplementary file 11 [file Image_2.TIFF]

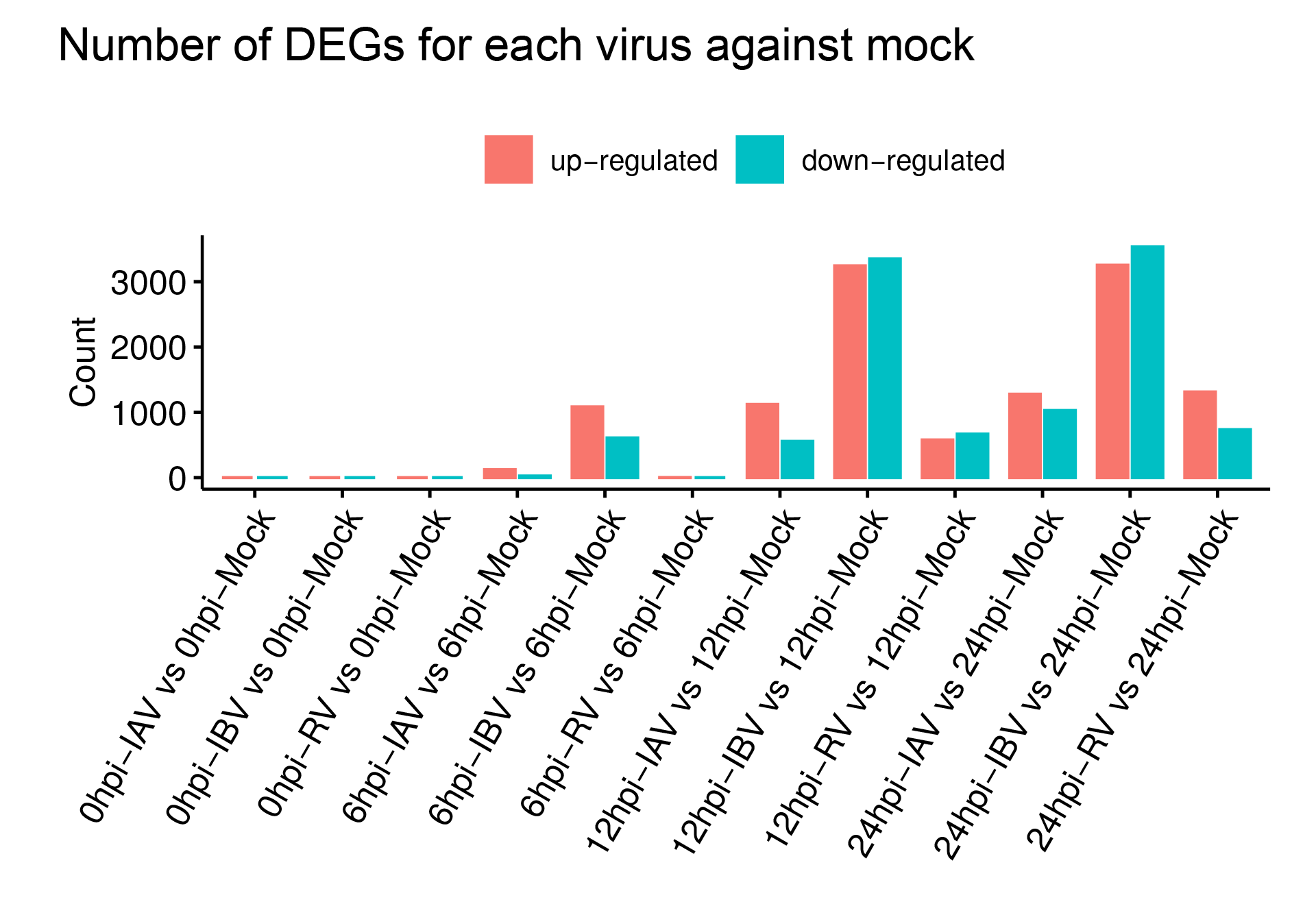

Supplement: Supplementary file 12 [file Image_3.TIF]

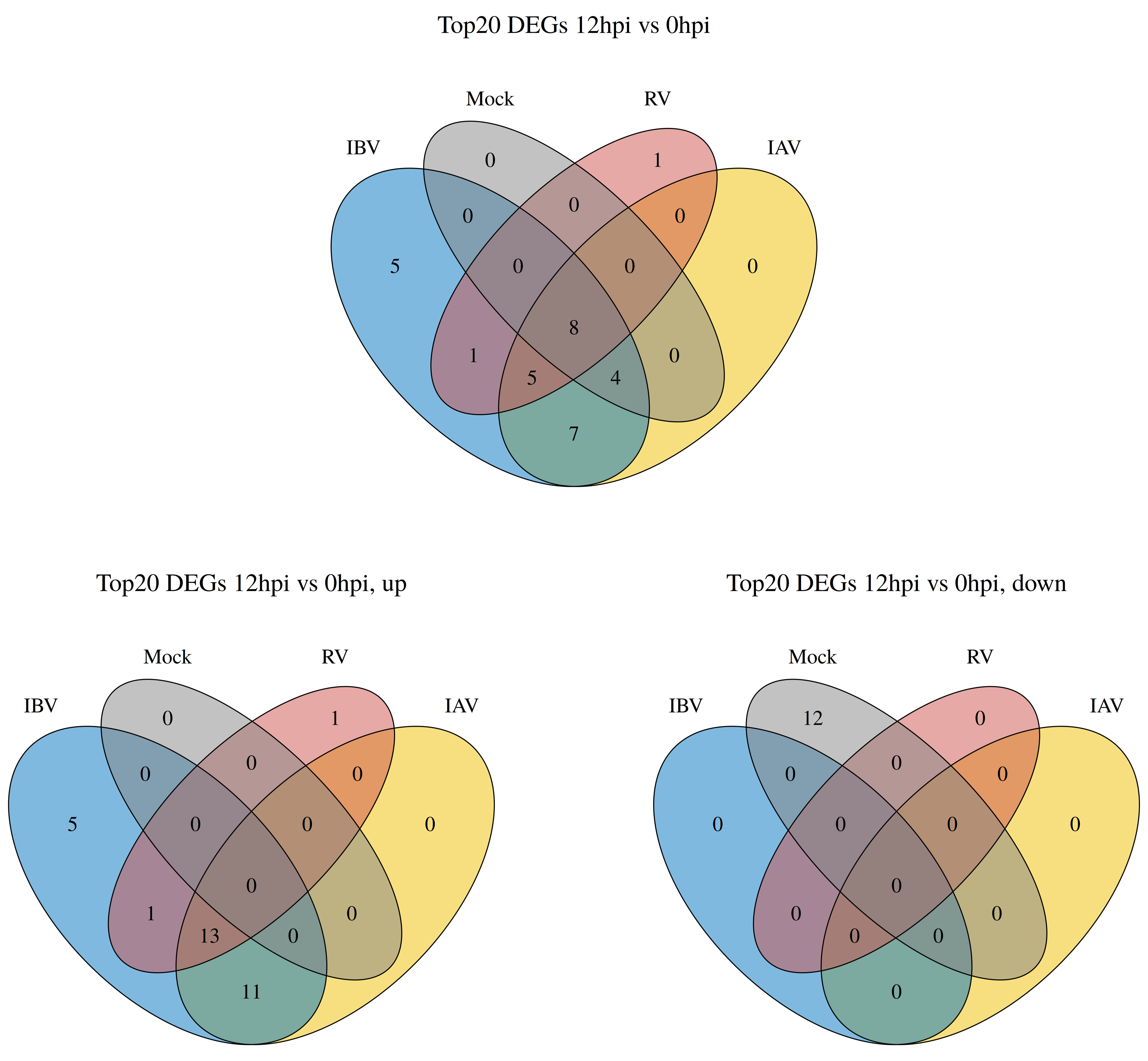

Supplement: Supplementary file 13 [file Image_4.PNG]

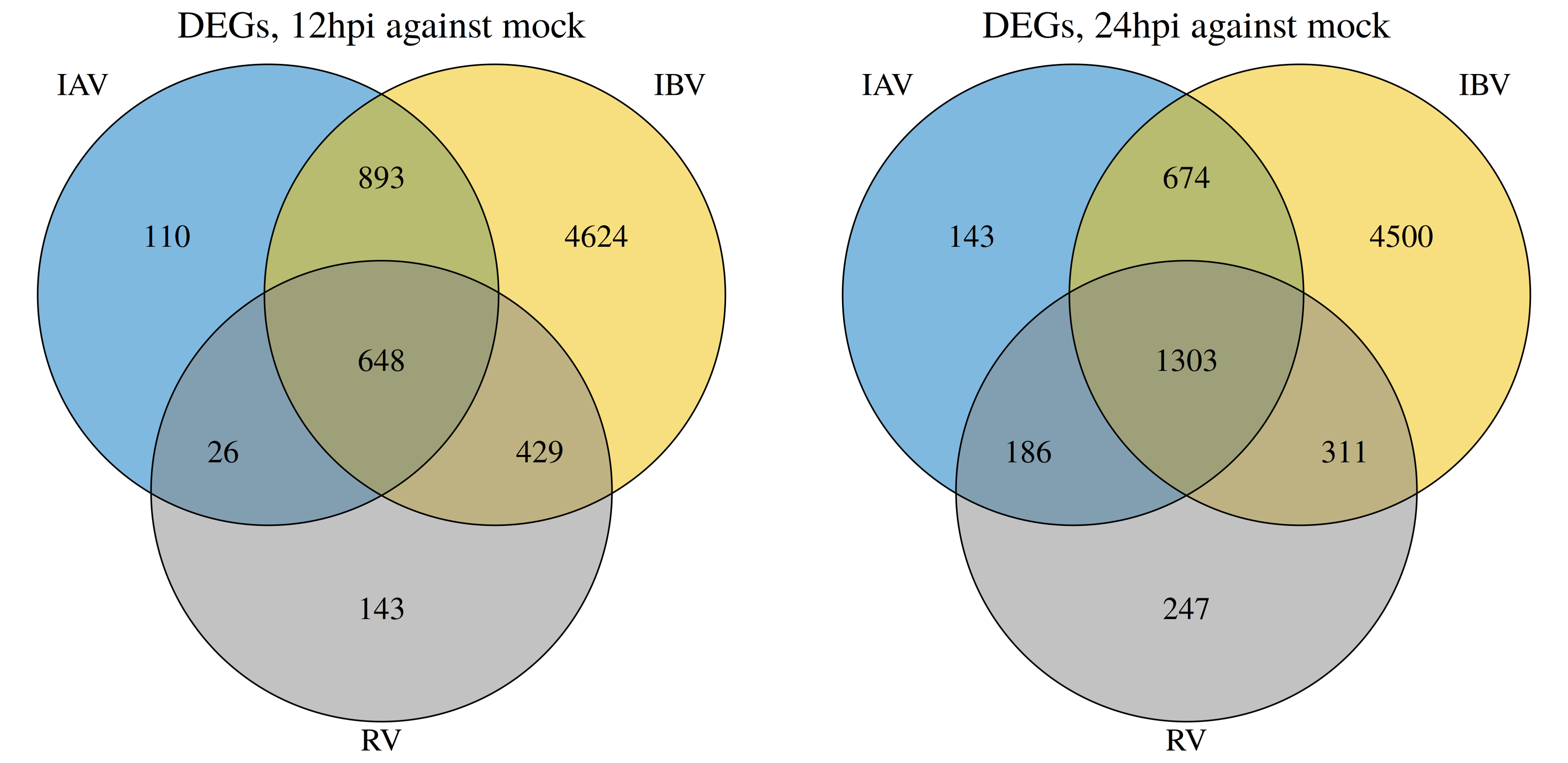

Supplement: Supplementary file 14 [file Image_5.PNG]

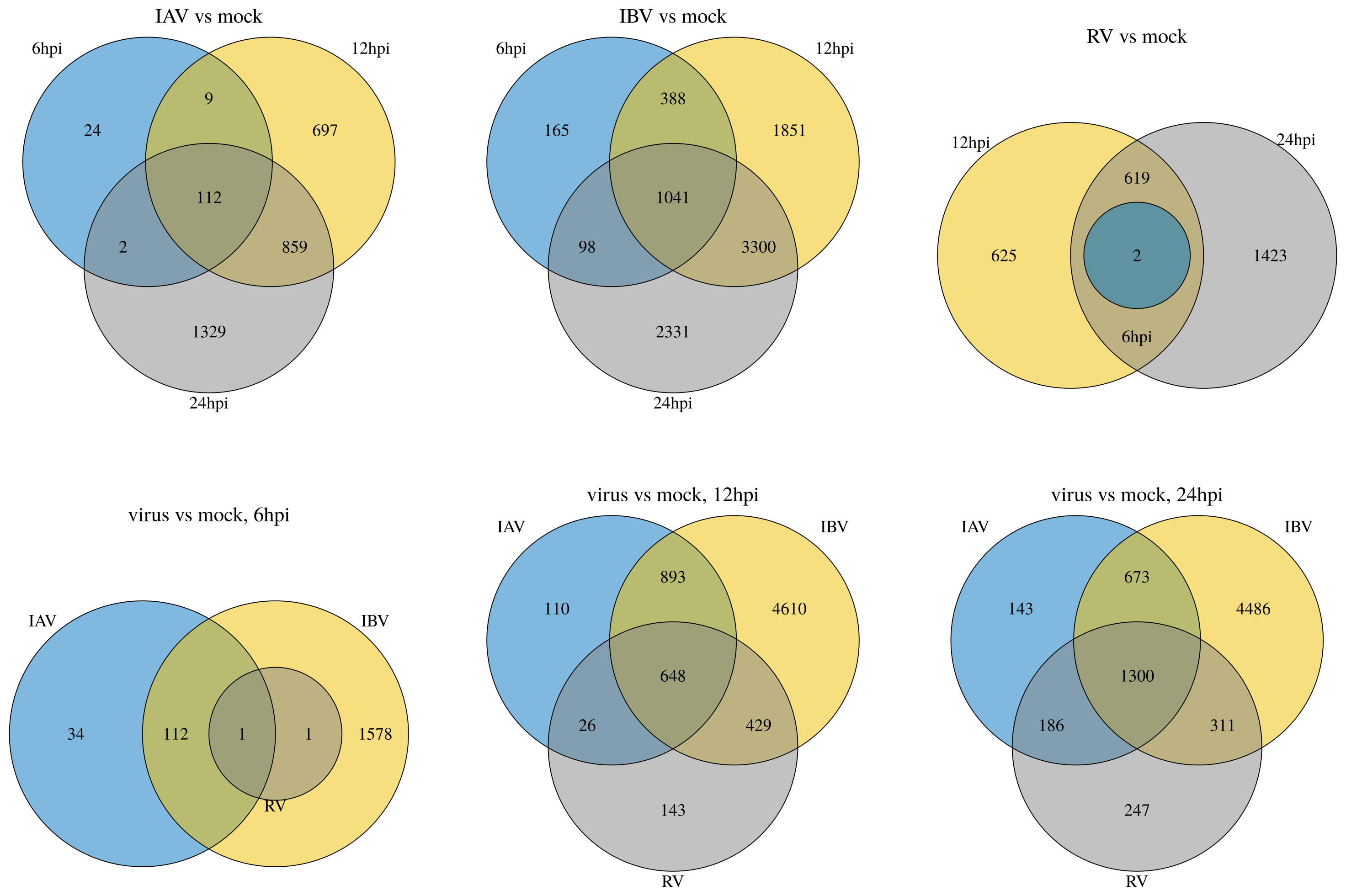

Supplement: Supplementary file 15 [file Image_6.PNG]
