# Supplementary material for: Comparative Transcriptomic Analysis of Rhinovirus and Influenza Virus Infection
Source: Front Microbiol. 2020 Jul 21;11:1580. doi: 10.3389/fmicb.2020.01580 (PMC7396524; doi:10.3389/fmicb.2020.01580)
Supplement: Supplementary file 16 [file Table_1.DOCX]

**Supplementary Table S1**. Primers and probes for real time RT-qPCR for influenza A virus, influenza B virus and rhinovirus

| **Virus** | **Primer/Probe** | **Primer/Probe sequence (5’-3’)** |
| --- | --- | --- |
| Influenza A virus | Forward | GACCRATCCTGTCACCTCTGAC |
|  | Reverse | AGGGCATTYTGGACAAAKCGTCTA |
|  | Probe | FAM/TGCAGTCCTCGCTCACTGGGCACG/3BHQ1 |
| Influenza B virus | Forward | ACAATTGCCTACYTGCTTTCA |
|  | Reverse | TCTTTCCCACCRAACCAAC |
|  | Probe | HEX-AGAAGATGGAGAARGCAAAGCAGAACTAGC-lABkFQ |
| Rhinovirus | Forward 1 | AGCCYGCGTGGCKGCC |
|  | Forward 2 | AGCCYGCGTGGTGCCC |
|  | Reverse | GAAACACGGACACCCAAAGTAGT |
|  | Probe | HEX-TCCGGCCCC-ZEN-TGAATGYGGCTAA-3IABkFQ |
