# Supplementary material for: Comparative Transcriptomic Analysis of Rhinovirus and Influenza Virus Infection
Source: Front Microbiol. 2020 Jul 21;11:1580. doi: 10.3389/fmicb.2020.01580 (PMC7396524; doi:10.3389/fmicb.2020.01580)
Supplement: Supplementary file 17 [file Table_2.DOCX]

**Supplementary Table S2**. Primers and probes for real time RT-qPCR for ICAM5, cytokines, chemokines and GAPDH

| Gene | Primer/Probe | Primer/Probe sequence (5’-3’) |
| --- | --- | --- |
| ICAM5 | Forward | TTGGCGCGGCAGCTGGT |
|  | Reverse | GCATCAGCTCTACGCGATCT |
|  | Probe | FAM/AGACTCAGC/ZEN/CCGTCTGCTTCTTCC/3IABkF |
| IL6 | Forward | GGCTGCAGGACATGACAACT |
|  | Reverse | ATCTGAGGTGCCCATGCTAC |
| CXCL10 | Forward | AGCAGAGGAACCTCCAGTCT |
|  | Reverse | ATGCAGGTACAGCGTACAGT |
| IFNβ | Forward | GCCGCATTGACCATCT |
|  | Reverse | CACAGTGACTGTACTCCT |
| GAPDH | Forward | ATTCCACCCATGGCAAATTC |
|  | Reverse | CGCTCCTGGAAGATGGTGAT |
| TNFα | Forward | CAAGGACAGCAGAGGACCAG |
|  | Reverse | TGGCGTCTGAGGGTTGTTTT |
