# Supplementary material for: Comparative Transcriptomic Analysis of Rhinovirus and Influenza Virus Infection
Source: Front Microbiol. 2020 Jul 21;11:1580. doi: 10.3389/fmicb.2020.01580 (PMC7396524; doi:10.3389/fmicb.2020.01580)
Supplement: Supplementary file 18 [file Table_3.DOC]

**Supplementary Table S3**. Quality control data from CGS. Distribution of throughput for each sample.

| **Sample Name** | **Number of Raw Reads (Read 1 + Read 2)** | **Total Throughput (Gb)** | **% of >= Q30 Bases** |
| --- | --- | --- | --- |
| 1Nil0 | 64,019,880 | 6.5 | 93 |
| 2Nil0 | 60,965,850 | 6.2 | 93 |
| 3FluA0 | 62,873,906 | 6.4 | 93 |
| 4FluA0 | 61,465,900 | 6.2 | 93 |
| 5FluB0 | 66,785,196 | 6.7 | 93 |
| 6FluB0 | 64,063,272 | 6.5 | 93 |
| 7Rhino0 | 64,832,884 | 6.5 | 93 |
| 8Rhino0 | 66,705,352 | 6.7 | 93 |
| 10Nil6 | 60,709,068 | 6.1 | 93 |
| 11FluA6 | 63,008,478 | 6.4 | 92 |
| 12FluA6 | 73,549,476 | 7.4 | 93 |
| 13FluB6 | 63,674,028 | 6.4 | 93 |
| 14FluB6 | 63,504,064 | 6.4 | 93 |
| 15Rhino6 | 70,071,826 | 7.1 | 93 |
| 16Rhino6 | 69,169,586 | 7.0 | 92 |
| 9Nil6 | 70,635,612 | 7.1 | 93 |
| 17Nil12 | 69,959,780 | 7.1 | 92 |
| 18Nil12 | 55,878,452 | 5.6 | 92 |
| 19FluA12 | 60,103,286 | 6.1 | 92 |
| 20FluA12 | 70,185,602 | 7.1 | 92 |
| 21FluB12 | 68,272,988 | 6.9 | 93 |
| 22FluB12 | 64,844,700 | 6.5 | 93 |
| 23Rhino12 | 64,783,104 | 6.5 | 93 |
| 24Rhino12 | 66,136,412 | 6.7 | 92 |
| 25Nil24 | 61,192,482 | 6.2 | 93 |
| 26Nil24 | 62,078,418 | 6.3 | 93 |
| 27FluA24 | 61,512,616 | 6.2 | 93 |
| 28FluA24 | 61,365,610 | 6.2 | 93 |
| 29FluB24 | 68,205,610 | 6.9 | 93 |
| 30FluB24 | 61,860,640 | 6.2 | 93 |
| 31Rhino24 | 70,644,010 | 7.1 | 93 |
| 32Rhino24 | 66,279,818 | 6.7 | 93 |
